# Recruitment and polarization typing of tumor-associated macrophages is associated with tumor progression and poor prognosis in Wilms tumor patients

**DOI:** 10.1371/journal.pone.0309910

**Published:** 2024-11-12

**Authors:** Zhang Wang, Liming Jin, Jinkui Wang, Xiaomao Tian, Tao Mi, Mujie Li, Zhaoxia Zhang, Xin Wu, Maoxian Li, Jiayan Liu, Zhaoying Wang, Yimeng Liu, Junyi Luo, Chunnian Ren, Dawei He

**Affiliations:** 1 Department of Urology, Children’s Hospital of Chongqing Medical University, National Clinical Research Center for Child Health and Disorders, Ministry of Education Key Laboratory of Child Development and Disorders, Chongqing, China; 2 Chongqing Key Laboratory of Pediatrics, Chongqing, China; Texas A&M University, UNITED STATES OF AMERICA

## Abstract

**Purpose:**

Tumor-associated macrophages (TAMs) play a crucial role in shaping various tumor microenvironments. However, their recruitment in Wilms tumor (WT), the predominant malignant renal tumor in children, has been inadequately explored. This retrospective cohort study involved the analysis of 148 WT samples to investigate the recruitment and polarization typing of TAMs in WT tissues.

**Methods:**

WT tissues underwent Western blotting (WB), reverse transcription-quantitative polymerase chain reaction (RT-qPCR), and immunofluorescence (IF) to measure the expression of TAM markers CD68, CD86, and CD163. Statistically analyze the relationship between TAM recruitment levels and patient clinical characteristics, and use Kaplan-Meier curves and the log-rank test to evaluate the association between TAM levels and survival outcomes.

**Results:**

The findings indicated a positive correlation between the recruitment levels of total macrophages (Mtotal) and M2 tumor-associated macrophages (M2 TAM) in both chemotherapy and non-chemotherapy groups with the clinical stage. Elevated recruitment of Mtotal and M2 TAM in tumor tissues was linked to a poorer prognosis. Notably, patients with persistently higher recruitment of M2 TAM following preoperative chemotherapy exhibited the worst prognosis.

**Conclusions:**

The recruitment and polarization typing of TAM exhibit significant differences in WT patients with various stages and prognosis outcomes, suggesting a potential avenue for future diagnosis and treatment of WT.

## 1. Introduction

Wilms tumor (WT) represents one of the most prevalent extracranial solid tumors affecting children, prompting considerable attention from the medical community. It constitutes over 90% of pediatric renal malignant tumors, predominantly afflicting children aged 2–4 years. Clinical presentations often manifest as a painless abdominal mass, occasionally accompanied by hematuria, fever, anemia, and additional symptoms [1]. Increasing research indicates that Wilms’ tumor originates from abnormal fetal kidney development, regulated by a complex network of environmental and genetic factors. Gene abnormalities in the ureteric bud during the fifth week of embryonic development and postnatal kidney tissues, such as WT1, SIX2, and CTNNB1, are significant contributors to the pathogenesis of Wilms’ tumor. The Children’s Oncology Group (COG) has stratified WT into stages I to IV based on disease progression and prognostic outcome, further categorizing it pathologically into good prognosis (FH) and poor prognosis (uFH) depending on the extent of intermediate cell infiltration within the tissue [1, 2].

In addition to surgical resection, the introduction of various adjuvant therapies, including chemotherapy, radiotherapy, targeted therapy, and immunotherapy, has markedly improved the prognosis of children with WT over the past half-century. Notably, individuals with low to moderate malignancy now exhibit a 5-year survival rate approaching 90% [2]. However, challenges persist in more intricate cases characterized by genetic pathogenic variants, recurrence, metastases, or poor tissue types with diffuse interstitial pathology [3]. The tumor microenvironment (TME) is a complex network composed of tumor cells, tumor-associated fibroblasts, immune cells, extracellular matrix, vascular system, and cytokines. It significantly impacts tumor growth, invasion, metastasis, and treatment resistance [4]. Poor prognosis in Wilms’ tumor (WT) is also profoundly influenced by the TME, making the investigation of WT’s tumor microenvironment a critical priority for effective treatment.

The regulation of various inflammatory immune responses and phagocytic repair by macrophages is a fundamental component of the tumor microenvironment (TME) [5]. Mononuclear macrophages, displaying high plasticity, undergo polarization by the TME during differentiation to transform into tumor-associated macrophages (TAMs), which play a significant role in modulating the TME [6]. Contemporary studies have categorized TAMs into two principal types: M1-type macrophages, known for promoting inflammation and anti-tumor responses, and M2-type macrophages, which suppress inflammation while fostering tissue remodeling and tumor growth. Both of these distinct TAM types have been observed in various tumor tissues, including Wilms tumor (WT) [7]. Nevertheless, the precise relationship between TAM polarization types, recruitment patterns within WT tumor tissues, and patient survival outcomes remains unclear. Elucidating the TAM-WT relationship could provide novel clues for TAM-targeted therapies in poor-prognosis WT.

This study examines the recruitment and polarization of TAMs in Wilms tumor (WT) tissues, analyzes their correlation with clinical characteristics in pediatric patients, and investigates the association between TAMs and the prognosis of children with WT. The research findings establish a foundation for the clinical diagnosis and treatment related to TAMs in WT.

## 2. Materials and methods

### 2.1 Patients and samples

A total of 148 Wilms tumor tissue samples with pathological diagnoses were collected between May 2000 and July 2022. The samples included 84 cases from patients who did not undergo chemotherapy before surgery (non-chemotherapy group) and 64 cases from those who received preoperative chemotherapy (chemotherapy group). Paraffin sections were prepared by the Pathology Department of Children’s Hospital Affiliated to Chongqing Medical University for subsequent experiments. Additionally, 8 frozen tumor tissue samples and their corresponding paracancerous tissues from Wilms tumor patients without preoperative chemotherapy were obtained from the biological tissue sample bank of the Children’s Hospital. Clinical data, including age, sex, surgical plan, pathological classification, tumor stage, and chemotherapy plan, were retrieved from patients’ hospital files. Conduct telephone follow-ups to gather information on patient prognosis, including survival outcomes, recurrence rates, myelosuppression, and tumor-related complications. Informed consent was obtained from all patients and/or their parents. The study was conducted in accordance with the Declaration of Helsinki, and approved by the Institutional Review Board of Children’s Hospital of Chongqing Medical University (IRB #2022–50).

The inclusion criteria for this study were as follows: (1) patients diagnosed with Wilms tumor (WT) at our hospital with well-defined pathological stage and histologic type; (2) availability of complete clinical data, including regular treatment and follow-up; (3) availability of paraffin-embedded tissue obtained after surgical treatment; (4) written informed consent from both the child and guardian. Exclusion criteria were as follows: (1) concurrent diagnosis of another tumor; (2) incomplete clinical data; (3) patients not receiving regular treatment.

### 2.2 Tissue total RNA extraction, reverse transcription and RT-qPCR

Tissue RNA was extracted using the TRIzol method (Invitrogen, Carlsbad, CA, USA). 100 μg of tissue was digested with 1 mL of TRIzol, homogenized, and mixed with chloroform. After centrifugation, the supernatant was combined with isopropanol, followed by ethanol precipitation. The obtained total RNA was vacuum-dried, dissolved in DEPC-treated water, and assessed for quality using NanoDrop ND-1000 spectrophotometer (D260/D280 between 1.8~2.0). Reverse transcription to cDNA was conducted using miRNA and mRNA reverse transcription kits (TaKaRa Company, Tokyo, Japan), and qRT-PCR was performed with designed primers (Shenggong Bioengineering, Shanghai, China). Primer sequences and annealing temperatures are detailed in Table 1.

**Table 1:** Primer sequence of tumor associated macrophage (TAM) marker.

TAM Marker	Primer sequence (5’-3’)	TM (°C)
CD163	Forward: ATCAACCCTGCATCTTTAGACA	60
	Reverse: CTTGTTGTCACATGTGATCCAG	
CD206	Forward: GACGTGGCTGTGGATAAATAAC	60
	Reverse: CAGAAGACGCATGTAAAGCTAC	
CD86	Forward: TGCTCATCTATACACGGTTACC	60
	Reverse: TGCATAACACCATCATACTCGA	
CD68	Forward: CCCAGATTCAGATTCGAGTCAT	60
	Reverse: GTTTTGTTGGGGTTCAGTACAG	
CD80	Forward: ACGGAGGCAGGGAACATCACC	60
	Reverse: GAAAGACCAGCCAGCACCAAGAG	
ARG-1	Forward: GTGGAAACTTGCATGGACAAC	60
	Reverse: AATCCTGGCACATCGGGAATC	

### 2.3 Tissue protein extraction and Western blot (WB)

Tissue lysates were prepared by digesting 100 μg of tissue in 1 ml RIPA with 100 μl PMSF. The lysate was homogenized for 1–2 min, followed by centrifugation at 4°C, 12,000 rpm for 20 min. The supernatant was used for BCA protein concentration determination, and protein samples (20 μg) were loaded onto SDS polyacrylamide gel. After separation, proteins were transferred to PVDF membranes, blocked, and incubated overnight at 4°C with primary antibodies (anti-CD163, anti-CD86, and anti-β-actin, 1:1000). Subsequently, membranes were washed, incubated with a secondary antibody (1:5000), and chemiluminescence was detected using ECL. Images were captured for analysis with Image Lab.

### 2.4 Immunofluorescence staining (IF)

Paraffin-embedded tissue sections (4 μm thickness) were dewaxed, subjected to high-temperature citrate antigen retrieval, and blocked with 0.5% BSA. They were then incubated overnight at 4°C with double fluorescent primary antibodies (rabbit CD163 + mouse CD68, rabbit CD86 + mouse CD68; Proteintech, Inc., Rosemont, IL, USA). After rewarming for 30 minutes, sections were incubated with rabbit and mouse double fluorescent secondary antibodies (FITC for green fluorescence, Cy3 for red fluorescence; Proteintech, Inc., Rosemont, IL, USA) for 1 hour at room temperature. Following PBS washing, DAPI staining was applied, and sections were sealed with an anti-fluorescent quencher agent.

### 2.5 Image acquisition and digital image analysis

Images were acquired using the VS200 Research Slide Scanner (Olympus, Tokyo, Japan) and the Nikon C2+ laser confocal microscope. Total cells were identified by nuclear staining with DAPI. CD68+ cells were considered total macrophages, CD68+CD163+ as M2 macrophages, and CD68+CD86+ as M1 macrophages. Three digital images (300 μm×600 μm) were randomly selected from each whole tissue section. Using Image J, the number of fluorescence signal-positive cells was counted, and the proportion to the total cell number was determined.

### 2.6 Statistical analysis

All data were analyzed and plotted using SPSS 21 (SPSS Inc., Chicago, IL, USA) and GraphPad Prism 10 (La Jolla, CA, USA) software. Student’s t-test and non-parametric tests were used to assess differences between two groups. One-way analysis of variance (ANOVA) was employed to evaluate differences among multiple groups. Kaplan-Meier analysis and Log-rank test were utilized for plotting patient survival curves and assessing differences. Non-normally distributed data were described using median (interquartile range) and differences were assessed using the Mann-Whitney test, with significance set at *p* < 0.05. Cox regression models were used for univariate and multivariate analyses, calculating odds ratios (OR) and 95% confidence intervals (CI), with significance set at *p* < 0.05. Overall survival (OS) was defined as the time from surgery to death or the last follow-up, with the last follow-up date for all patients being November 27, 2023.

## 3. Results

### 3.1 Patient characteristics

This study incorporated a total of 148 paraffin sections from Wilms tumor (WT) specimens, comprising 84 patients who had not undergone chemotherapy before surgery (non-chemotherapy group) and 64 patients subjected to preoperative chemotherapy (chemotherapy group). In the non-chemotherapy group, there were 38 males and 46 females, with a median age of 33.5 months (range: 2–151 months). Among them, 44 patients were younger than 36 months, and 40 patients were older than 36 months. Clinical staging, employing the Children’s Oncology Group (COG) stage, revealed 6 patients in stage I, 27 in stage II, 31 in stage III, and 20 in stage IV. In the chemotherapy group, there were 23 males and 41 females, with a median age of 36 months (range: 9–132 months). Among them, 33 patients were younger than 36 months, and 31 patients were older than 36 months. Clinical staging identified 4 patients in stage II, 28 in stage III, and 32 in stage IV (Table 2).

**Table 2:** Clinical information of 148 WT patients.

Clinical features		Non-chemotherapy	Chemotherapy
		Number of patients (%)	Number of patients (%)
Age(month)	≤36	44(52.4)	33(51.6)
	>36	40(47.6)	31(48.4)
Gender	Male	38(45.2)	23(35.9)
	Female	46(54.8)	41(64.1)
Stage (COG)	Ⅰ	6(7.1)	0(0)
	II	27(32.2)	4(6.2)
	III	31(36.9)	28(43.8)
	IV	20(23.8)	32(50)
Pathological type	FH	73(86.9)	56(87.5)
	uFH	11(13.1)	8(12.5)
Death		10(11.9)	14(21.9)
Total		84(100)	64(100)

### 3.2 Increased expression of tumor-associated macrophage markers in Wilms tumor tissue

**Fig 1:**
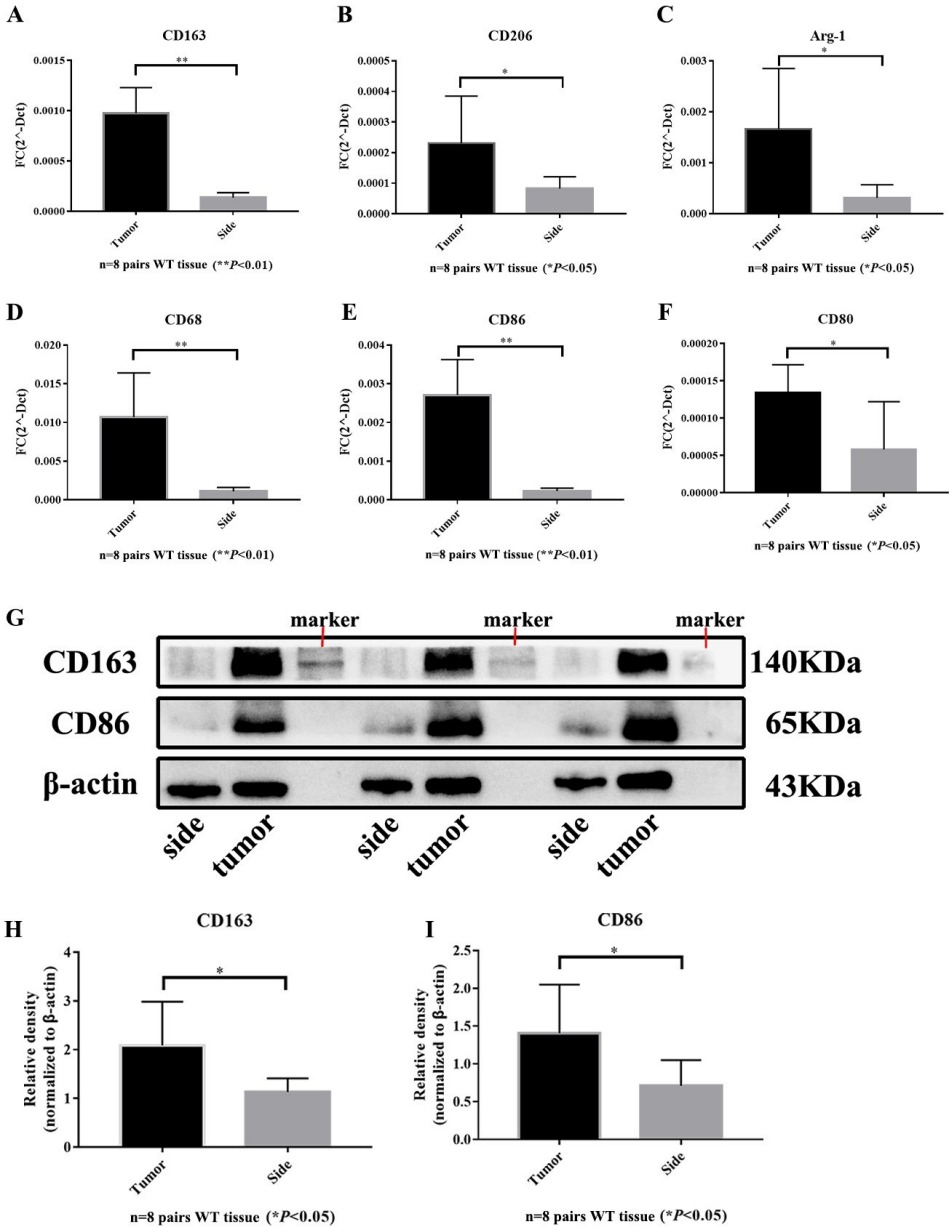
Recruitment of TAM in WT and paracancerous tissue. (A-C) mRNA expression of M2 TAM markers; (D) mRNA expression of total macrophage marker; (E-F) mRNA expression of M1 TAM markers (*p<0.05; **p<0.01; *p*<0.05 was considered statistically significant).

In this study, we included 8 cases of Wilms tumor frozen tissue and corresponding paracancerous tissues. The expression of tumor-associated macrophage markers was assessed using quantitative polymerase chain reaction (qPCR) and Western blot (WB). The statistical analysis indicated that the expression of the general macrophage marker CD68 (Fig 1D) was higher in tumor tissues compared to adjacent tissues. Additionally, the expression of M1 macrophage markers CD86 and CD80 (Fig 1E and 1F) in tumor tissues surpassed that in adjacent tissues, while the expression of M2 macrophage markers CD163, CD206, and Arg-1 in tumor tissues was also higher than in adjacent tissues (*p*<0.05, *p*<0.01) (Fig 1A–1C). Western blot results further demonstrated that the protein expression of M1 and M2 macrophage markers CD86 and CD163 in tumor tissues was significantly higher than in paracancerous tissues (*p*<0.05) (Fig 1G–1I). These findings collectively reveal the recruitment of tumor-associated macrophages by Wilms tumor tissues.

### 3.3 Correlation of tumor-associated macrophages with clinical features of the Wilms tumor

**Fig 2:**
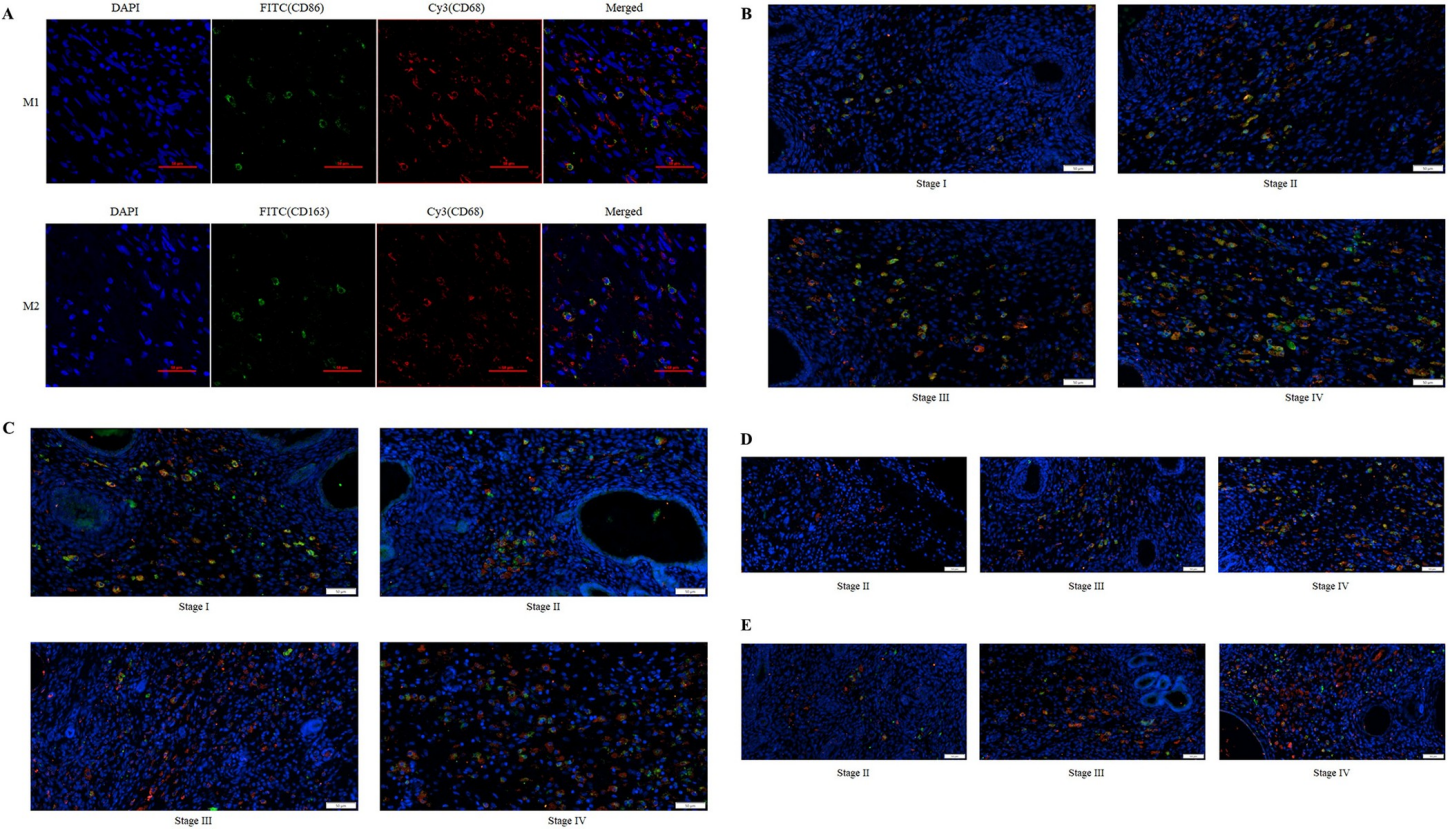
IF detection of TAM recruitment in Wilms tumor tissue. (A) Representative immunofluorescence images of CD68, CD163, and CD86 staining in WT tissue sections; (B) Recruitment of CD68+CD163+M2 macrophages in Wilms tumor of non-chemotherapy group; (C) Recruitment of CD68+CD86+M1 macrophages in Wilms tumor of non-chemotherapy group; (D) Recruitment of CD68+CD163+M2 macrophages in Wilms tumor of chemotherapy group; (E) Recruitment of CD68+CD86+M1 macrophages in Wilms tumor of chemotherapy group (Red: overall macrophage Mtotal; Green: Figure B and D are M2 macrophages, Figure C and E are M1 macrophages) (50 μm Ruler).

To further elucidate the relationship between tumor-associated macrophages (TAMs) and Wilms tumor, immunofluorescence staining was employed to investigate TAM recruitment and typing in tumor tissues. The number of nuclei exhibiting positive staining with DAPI (blue) was designated as total cells, and CD68 (Cy3 red fluorescence) + CD86/CD163 (FITC green fluorescence) double fluorescent staining was utilized to label tumor-associated macrophages (Fig 2A). Specifically, CD68+ cells were defined as total macrophages (Mtotal), CD68+CD163+ as M2-type macrophages (Fig 2B and 2D), and CD68+CD86+ as M1-type macrophages (Fig 2C and 2E).

In Fig 2, the general macrophage marker CD68 was predominantly observed in the loose regions of Wilms tumor tissue. The fluorescence expression of the M2 marker CD163 increased with the elevation of tumor stage, whereas the expression of the M1 marker CD86 was elevated in tumor tissues with lower tumor stages. Following chemotherapy, the expression of all three fluorescences in tumor tissues exhibited a decrease. Subsequently, our focus will be on quantifying macrophages of different polarization types in Wilms tumor tissue.

### 3.4 Association of TAM with clinical features in patients without preoperative chemotherapy

**Fig 3:**
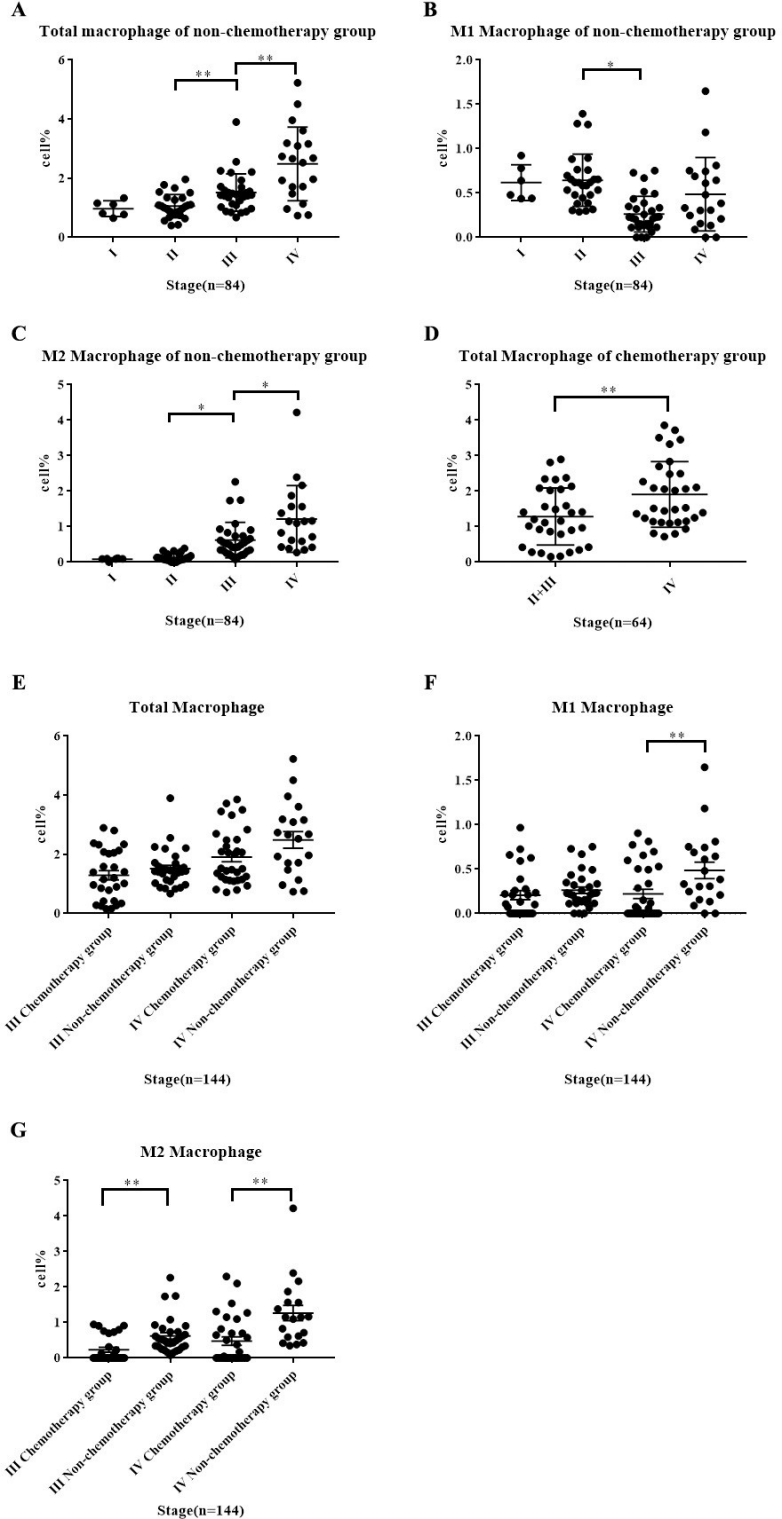
The recruitment difference of TAM in different stages WT tissue. (A-C) The recruitment difference of TAM in different stages WT in the non-chemotherapy group; (D) The recruitment difference of overall macrophage Mtotal in WT tissue of the chemotherapy group; (E-G) The recruitment difference of TAM in WT tissues between non-chemotherapy and chemotherapy group during the same stage (*p<0.05; **p<0.01; *p*<0.05 was considered statistically significant).

The results revealed that the median Mtotal% in the tissues of Wilms tumor in the non-chemotherapy group was 1.385 (0.949, 1.732), the median M1% was 0.411 (0.218, 0.654), and the median M2% was 0.336 (0.114, 0.721). The recruitment of Mtotal and M2 tumor-associated macrophages (TAMs) exhibited a positive correlation with clinical stage, with the recruitment of M2 TAMs in stage III and IV tumor tissues increasing sequentially (*p*<0.01). Conversely, M1 TAMs decreased significantly in stage III tumor tissues, with no statistical differences observed in stages I, II, and IV. Due to the small sample size of the poor prognostic type (uFH), the recruitment of Mtotal/M1/M2 macrophages did not show a difference between the good prognosis type (FH). Additionally, there were no significant differences in TAM recruitment based on patient age and sex (Table 3 and Fig 3A–3C).

**Table 3:** The recruitment difference of TAM in WT of non-chemotherapy group.

Clinical features		n	Mtotal			M1		M2		
			Medium%	*p*		Medium%	*p*	Medium%	*p*	
Age(month)	≤36	44	1.29(0.96, 1.57)		0.6499	0.44(0.25, 0.66)	0.3247	0.23(0.09, 0.50)		0.0687
	>36	40	1.40(0.94, 1.96)			0.37(0.16, 0.65)		0.42(0.20, 0.91)		
Gender	Male	38	1.35(0.88, 1.89)		0.6957	0.38(0.25, 0.72)	0.3081	0.38(0.16, 0.89)		0.6442
	Female	46	1.37(0.97, 1.69)			0.44(0.21, 0.62)		0.32(0.09, 0.61)		
Stage	I	6	0.97(0.79, 1.15)		<0.01	0.56(0.45, 0.75)	<0.01	0.09(0.08, 0.10)		<0.01
	II	27	1.00(0.82, 1.30)			0.62(0.46, 0.73)		0.10(0.06, 0.19)		
	III	31	1.44(1.11, 1.64)			0.22(0.13, 0.34)		0.50(0.32, 0.74)		
	IV	20	2.59(1.65, 3.17)			0.36(0.20, 0.70)		1.12(0.55, 1.57)		
Pathological type	FH	73	1.33(0.97, 1.78)		0.9925	0.44(0.21, 0.65)	0.9772	0.32(0.22, 0.82)		0.9967
	uFH	11	1.40(0.79, 1.48)			0.38(0.30, 0.56)		0.55(0.22, 0.82)		

M (QL, QU): Median (lower quartile, upper quartile); *p*<0.05 was considered statistically significant.

### 3.5 Association of TAM with clinical features in patients with preoperative chemotherapy

The results indicated that the median Mtotal% of Wilms tumor in the chemotherapy group was 1.411 (0.959, 2.113), with 32 cases (50%) showing the presence of M1 macrophages (M1 Positive), and a median positive M1% of 0.313 (0.146, 0.634). M2 macrophages (M2 Positive) were detected in only 27 cases (42.19%), with a median positive M2% of 0.731 (0.440, 1.023). Given the limited number of cases in stage II (only 4 cases), a t-test comparing stage III with stage IV demonstrated that the recruitment of overall macrophages (Mtotal) in tumor tissues after chemotherapy remained positively correlated with the clinical stage, where stage IV Mtotal was significantly higher than stage II+III (*p* = 0.0054) (Table 4, Fig 3D). The positive rates of M1 and M2 in stage II to IV after chemotherapy were tested using Fisher’s precision probability test (as more than half of the patients in the chemotherapy group expressed a TAM amount of 0, the Fisher’s precision probability test was employed here for counting data statistics), revealing no statistical differences (*p* values of 0.135 and 0.154, respectively). Considering the potential impact of chemotherapy drugs on the recruitment of tumor-associated macrophages, the sample size of unfavorable histology (uFH) in the chemotherapy group was too small, and no differences in Mtotal/M1/M2 macrophage recruitment were observed between FH and uFH tissues. Furthermore, there were no significant differences in TAM recruitment based on patient age and sex in the chemotherapy group (Table 5).

**Table 4:** The recruitment difference of overall macrophage Mtotal in WT tissues of the chemotherapy group.

Clinical features		n	Mtotal	
			Medium%	*p*
Age(month)	≤36	33	1.48(1.09, 2.34)	0.4704
	>36	31	1.40(0.95, 2.08)	
Gender	Male	23	1.41(0.95, 2.10)	0.1714
	Female	41	1.39(0.92, 2.09)	
Stage	II+III	32	1.18(0.70, 2.03)	0.0054
	IV	32	1.52(1.15, 2.49)	
Pathological type	FH	56	1.39(0.93, 2.17)	0.9902
	uFH	8	1.51(1.35, 2.04)	

M (QL, QU): Median (lower quartile, upper quartile); *p*<0.05 was considered statistically significant.

**Table 5:** The recruitment difference of M1 and M2 in WT tissue of the chemotherapy group.

Clinical features		n	M1			M2		
			Positive	Negative	*p*	Positive	Negative	*p*
Age(month)	≤36	33	17	16	0.802	14	19	0.968
	>36	31	15	16		13	18	
Gender	Male	23	9	14	0.193	12	11	0.226
	Female	41	23	18		15	26	
Stage	II	4	0	4	0.135	0	4	0.154
	III	28	16	12		11	17	
	IV	32	16	16		16	16	
Pathological type	FH	56	29	27	0.705	25	31	0.503
	uFH	8	3	5		2	6	

*p* <0.05 was considered statistically significant.

### 3.6 Differences in macrophage expression between non-chemotherapy group and chemotherapy group

The comparison between the non-chemotherapy group and the chemotherapy group revealed no significant difference in overall macrophage (Mtotal) recruitment in tumor tissues. However, the chemotherapy group exhibited varying degrees of decreased TAM recruitment. Notably, there were statistically significant reductions in stage IV M1 TAM (*p* = 0.0101), stage III M1 TAM (*p* = 0.012), and stage IV M2 TAM (*p* <0.01). These findings suggest a potential association with the impact of chemotherapy drugs. The inhibitory effect of chemotherapy drugs on TAMs, as reported in the literature, is reflected in the results of this experiment (Fig 3E–3G).

### 3.7 Relationship between TAM distribution and patient survival

**Fig 4:**
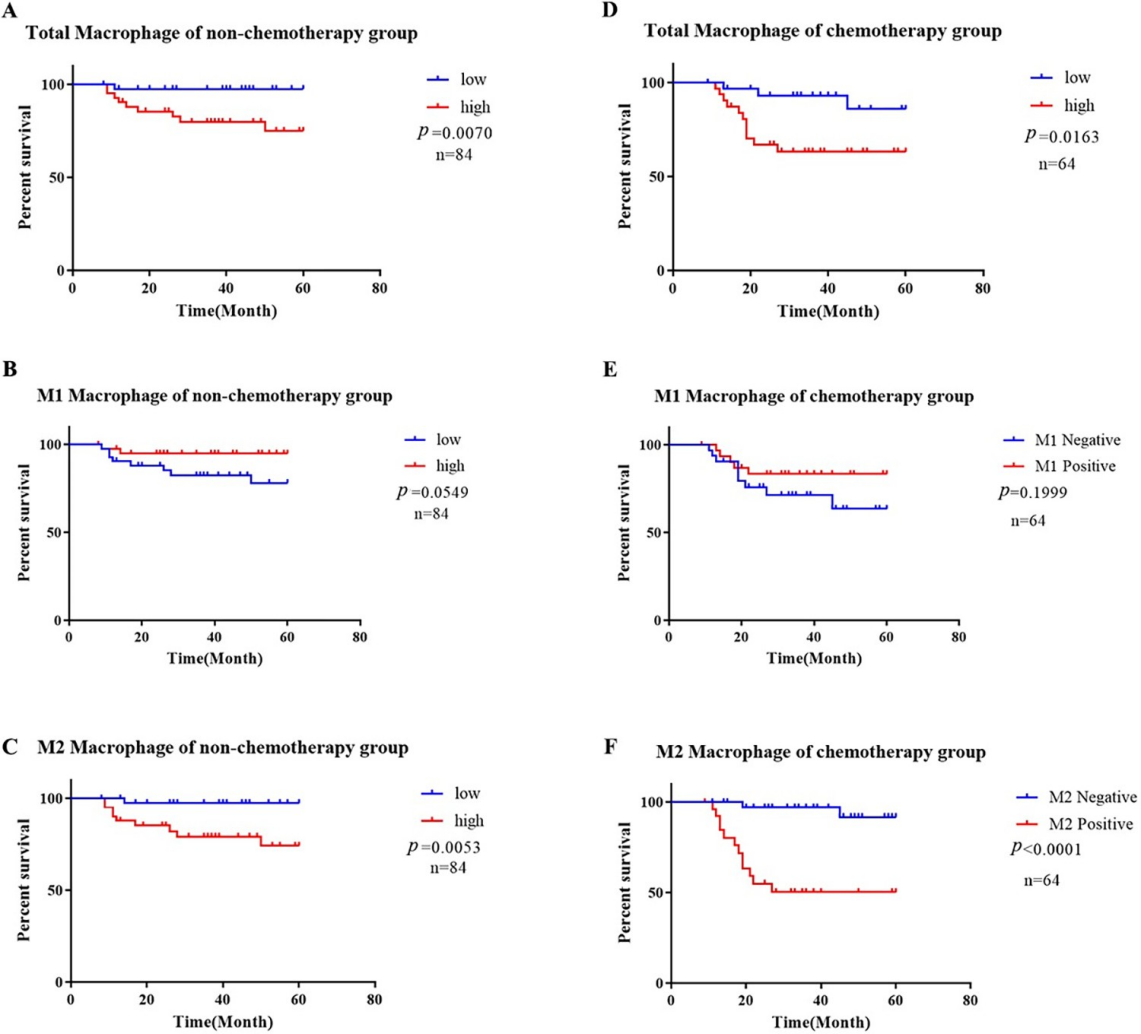
Survival curve of WT. (A-C) The relationship between TAM recruitment and prognosis of WT patients in non-chemotherapy group; (D-F) The relationship between TAM recruitment and prognosis of WT patients in chemotherapy group (*p*<0.05 was considered statistically significant).

A 5-year overall survival analysis was conducted using Kaplan-Meier analysis and the log-rank test to examine the association between macrophages and patient survival outcomes. Kaplan-Meier curves can illustrate the relationship between survival time and survival rate under specific conditions, while the log-rank test can evaluate whether there is a significant difference between the survival curves of two groups. In the non-chemotherapy group, the 5-year overall survival rate (OS) was 88.1% (74/84), with breakdowns by clinical stage as follows: 100% (6/6) in stage I, 96.3% (26/27) in stage II, 90.3% (28/31) in stage III, 70% (14/20) in stage IV. For the chemotherapy group, the 5-year survival rate was 78.1% (50/64), including 100% (4/4) in stage II, 85.7% (24/28) in stage III, and 68.8% (22/32) in stage IV. In the non-chemotherapy group, the 5-year survival rate for macrophage Mtotal was 97.6% (41/42) in the low-recruitment group and 78.6% (33/42) in the high-recruitment group. Similarly, for M2 macrophages, the 5-year survival rate was 97.6% (41/42) in the low-recruitment group and 78.6% (33/42) in the high-recruitment group. The log-rank test indicates that the recruitment of Mtotal and M2 TAM was significantly negatively correlated with patient survival (*p*<0.01), indicating that high levels of Mtotal and M2 TAM recruitment in the non-chemotherapy group predict a worse prognosis (Fig 4A–4C). In the chemotherapy group, the 5-year survival rate for macrophage Mtotal was 90.6% (3/32) in the low-recruitment group and 65.6% (11/32) in the high-recruitment group. The 5-year survival rate for M2 macrophages was 94.6% (35/37) in the low-recruitment group and 55.6% (15/27) in the high-recruitment group. The log-rank test indicates that the recruitment of Mtotal and M2 TAM in the chemotherapy group was still significantly negatively correlated with the survival rate of patients (*p*<0.05) (Fig 4D–4F). Notably, the Kaplan-Meier curve shows that the survival rate of patients with high M2 TAM recruitment in the chemotherapy group (55.6%) is significantly lower than that of patients with high M2 TAM recruitment in the non-chemotherapy group (78.6%)(Fig 4C and 4F).However, the recruitment of M1 was positively correlated with patient survival in both the chemotherapy and non-chemotherapy groups, although the trend did not reach statistical significance (*p* = 0.549, *p* = 0.1999). Expanding the sample size further may potentially accentuate these differences and bring out statistically significant trends. (Fig 4B and 4E).

To further analyze the relationship between TAMs and the prognosis of WT patients, we conducted a COX regression analysis. COX regression analysis examines the impact of various risk factors on survival outcomes, allowing us to determine whether TAMs are a significant risk factor for patient mortality through the regression model. COX multivariate regression analysis showed that M2 TAM recruitment in both non-chemotherapy (HR = 1.976, 95%CI = 1.313–2.972, *p* = 0.001) and chemotherapy (HR = 4.876, 95%CI = 2.538–9.37, *p* <0.001) group is factor in the poor prognosis of patients. Univariate regression analysis showed that the recruitment of total macrophages Mtotal (HR = 1.891, 95%CI = 1.237–2.892, *p* = 0.003) in the non-chemotherapy group was a poor prognostic factor, while the recruitment of M1 TAM (HR = 0.009, 95%CI = 0–0.322, *p* = 0.01) was a good prognostic factor. The recruitment of total macrophages Mtotal in the chemotherapy group (HR = 3.108, 95%CI = 1.664–5.806, *p*<0.001) was also a factor in the poor prognosis of patients (Tables 6 and 7).

**Table 6:** COX multivariate regression analyzed prognostic factors in WT patients of non-chemotherapy group.

Clinical features		Univariable			Multivariable		
		HR	95%CI	*p*	HR	95%CI	*p*
Age		1.007	0.99–1.024	0.419			
Sex	F	reference					
	M	1.16	0.336–4.007	0.815			
Stage	I+II	reference					
	III+IV	6.229	0.788–49.242	0.083			
Histological type	FH	reference					
	uFH	0.748	0.095–5.908	0.783			
Mtotal%		1.891	1.237–2.892	0.003			
M1%		0.009	0–0.322	0.01	0.03	0.001–1.333	0.07
M2%		2.415	1.652–3.532	<0.001	1.976	1.313–2.972	0.001

*p* <0.05 was considered statistically significant.

**Table 7:** COX multivariate regression analyzed prognostic factors in WT patients of chemotherapy group.

Clinical features		Univariable			Multivariable		
		HR	95%CI	*p*	HR	95%CI	*p*
Age		0.999	0.978–1.02	0.931			
Sex	F	reference					
	M	1.441	0.5–4.157	0.499			
Stage	II+III	reference					
	IV	3.039	0.952–9.7	0.061			
Histological type	FH	reference					
	uFH	1.133	0.253–5.079	0.87			
Mtotal%		3.108	1.664–5.806	<0.001			
M1%		1.129	0.161–7.894	0.903			
M2%		4.876	2.538–9.378	<0.001	4.876	2.538–9.37	<0.001

*p* <0.05 was considered statistically significant.

## 4. Discussion

The recruitment of tumor-associated macrophages (TAMs) is a significant contributor to the metastasis and treatment resistance observed in many tumors [8, 9]. However, their recruitment and polarization typing in Wilms tumor (WT) have remained unclear. This study confirmed the recruitment of TAMs in WT tissues, revealing that the recruitment of overall macrophages (Mtotal) and M2 TAMs in tumor tissues of both preoperative chemotherapy and non-chemotherapy patients was positively correlated with the stage of WT. Additionally, increased recruitment of Mtotal and M2 TAMs in tumor tissues of both preoperative chemotherapy and non-chemotherapy patients was associated with a poor prognosis. Furthermore, the prognosis was notably worse in patients with high recruitment of M2 TAMs after chemotherapy.

Tumor-associated macrophages (TAMs), a subset of macrophages, play a crucial role in regulating the initiation, progression, and metastasis of various cancers, including ovarian, breast, and colorectal cancers [10–12]. Macrophages exhibit strong plasticity and can differentiate into M1 and M2 phenotypes primarily in response to stimuli in the tumor microenvironment [13]. M1 TAMs promote inflammation by secreting cytokines such as IL-1β, inducible nitric oxide synthase (iNOS), and tumor necrosis factor-alpha (TNF-α) [14], exerting anti-tumor effects across multiple cancers [15, 16]. In contrast, M2 TAMs suppress inflammation while promoting tissue repair, remodeling, and tumor angiogenesis through the release of vascular endothelial growth factor (VEGF), epidermal growth factor (EGF), and fibroblast growth factor (FGF). Moreover, M2 TAMs support tumor cell proliferation, metastasis, and immune suppression [17–19], contributing to poor patient prognosis [20]. Our qPCR and Western blot results indicate elevated levels of total macrophages (Mtotal), M1, and M2 TAMs in Wilms tumor (WT) tissues compared to adjacent non-cancerous tissues, suggesting a significant infiltration of TAMs in WT tissues that may correlate with patient outcomes. However, the polarization of TAMs varies significantly among different tumors and even within different states of the same tumor. Therefore, gaining a clear understanding of WT, and specifically analyzing the relationship between WT staging, prognosis, and TAM polarization, is a critical next step in our research efforts.

Wilms tumor (WT) is the predominant malignant kidney tumor in children, accounting for 90% of pediatric renal tumors. It is also one of the most common extracranial solid tumors in children. Previous studies have noted the recruitment of mononuclear macrophages [21, 22], but specific TAM polarization analysis in WT tissues has been lacking. Through immunofluorescence analysis of 148 cases of WT tissues treated with or without preoperative chemotherapy at our hospital, we found that the expression of Mtotal and M2 macrophages in WT tissues was positively correlated with the clinical stage of malignancy in both non-chemotherapy and chemotherapy groups. Additionally, Mtotal and M2 macrophages were negatively correlated with overall survival rates in both patient groups. Piera Filomena Fiore et al. conducted a study investigating the interaction between primary Wilms tumor and immune cells. Their findings revealed that Wilms tumor can compromise the function of natural killer (NK) cells by promoting the polarization of M2 macrophages [23]. This underscores the promoting effect of M2 macrophages on the tumor microenvironment. Examining the role of overall macrophages in various tumors, such as ovarian cancer, medulloblastic brain tumors and others [24, 25], has unveiled a consistent correlation between high macrophage recruitment levels and favorable prognosis in children. In contrast, in certain tumors like Wilms tumor and follicular lymphoma, high levels of CD68+ macrophages are associated with poor prognosis [26]. Scholars attribute these divergent correlations to the different subcategories within macrophages [27], emphasizing the varying proportions of tumor-associated macrophages (TAMs). Accumulating evidence supports the cancer-promoting role of TAMs in diverse cancers, including renal cell carcinoma [28], glioblastoma [29], pancreatic cancer [30], head and neck cancer [31], and lymphoma [32]. A high proportion of TAMs is consistently linked to advanced tumor stage and shorter survival time [33]. COX multivariate regression analysis showed that the recruitment of M2 TAM was a poor prognostic factor in both chemotherapy and non-chemotherapy groups, and univariate analysis showed that the poor prognosis of patients in chemotherapy and non-chemotherapy groups was also associated with high recruitment level of overall macrophage Mtotal, and the increase in M1 TAM recruitment in the non-chemotherapy group suggested that patients had a better prognosis. In the present research, the recruitment of M2 TAMs exhibited a more significant trend difference than that of M1 type. Obviously, TAMs, especially the M2 type, have the potential to serve as prognostic indicators for predicting tumor malignancy and patient outcomes.

Compared to the non-chemotherapy group, the recruitment of TAMs in WT tissues is significantly reduced in the chemotherapy group, particularly the M2 type TAMs, indicating an evident effect of chemotherapy drugs. Commonly used preoperative chemotherapy for WT involves vincristine, actinomycin D, doxorubicin, cyclophosphamide, carboplatin and etoposide [34]. Studies have indicated that doxorubicin intervention in mouse tumor cells induces the release of ATP, leading to the recruitment of mononuclear phagocytes and differentiation of bone marrow cells into antigen-presenting cells, triggering an effective adaptive immune response [35]. Actinomycin D can also contribute to tumor cell destroying by promoting the transformation of monocyte macrophages toward an anti-tumor direction through drug-dependent cytotoxicity. The research findings demonstrated that the 5-year survival rate for cases with detectable tumor-promoting M2 macrophage residue after chemotherapy was notably lower at 40.7%, contrasting with the 5-year survival rate of 86.6% for M2-negative cases after chemotherapy. Besides mitigating the tumor toxicity of chemotherapy drugs such as platinum, paclitaxel, and doxorubicin [36, 37], residual M2 macrophages after chemotherapy can exhibit characteristics of ordinary monocytes-macrophages, promoting proliferation and tissue repair [38]. This, in turn, contributes to immune escape, drug resistance, and tumor growth and metastasis [39]. Additionally, residual M2-type TAMs aggregate in the perivascular region through the CXCR4/CXCL12 pathway, secreting factors like VEGF to further facilitate tumor revascularization and recurrent metastasis [40].

It’s important to note that the 5-year overall survival (OS) of patients in the chemotherapy group in this study was 78.1%, lower than the 5-year OS of patients in the non-chemotherapy group (88.1%). This seemingly counterintuitive results arise from that patients undergoing chemotherapy typically have larger tumor sizes and more rapid disease progression. Additionally, the chemotherapy group selected for this study predominantly included patients with advanced stages, with the majority being stage III and above, and only 4 cases below stage III. This selection bias in favor of patients with higher stages is a crucial factor contributing to these results. As a single-center pediatric oncology study, collecting a sufficient number of WT samples with complete clinical information and usable specimens was indeed challenging. However, considering the unavoidable selection bias in retrospective pathological control studies, we plan to use larger sample sizes in future prospective cohort studies to validate our findings.

Based on the above, we have reason to believe that TAMs hold significant value in the diagnosis and treatment of tumors. Current clinical therapies targeting TAMs are still under exploration [41]. How to reduce the recruitment of TAMs in WT tissues and even reverse M2 TAMs to M1 phenotype for anti-tumor effects are topics we will deepen our research into.

## 5. Conclusions

In summary, tumor-associated macrophages (TAMs) are of significant importance for the diagnosis and treatment of Wilms tumor (WT). This study has identified a high recruitment of TAMs in WT tissues. The recruitment of total macrophages (Mtotal) and M2 TAMs is positively correlated with the staging of WT patients and is a factor associated with poor prognosis. Additionally, WT patients with high M2 TAM recruitment in tumor tissues after chemotherapy exhibit the worst prognosis.

## Supporting information

S1 Data (ZIP)

S2 Data (ZIP)
