# Supplementary figures and images for: Recruitment and polarization typing of tumor-associated macrophages is associated with tumor progression and poor prognosis in Wilms tumor patients
Source: PLoS One. 2024 Nov 12;19(11):e0309910. doi: 10.1371/journal.pone.0309910 (PMC11556688; doi:10.1371/journal.pone.0309910)

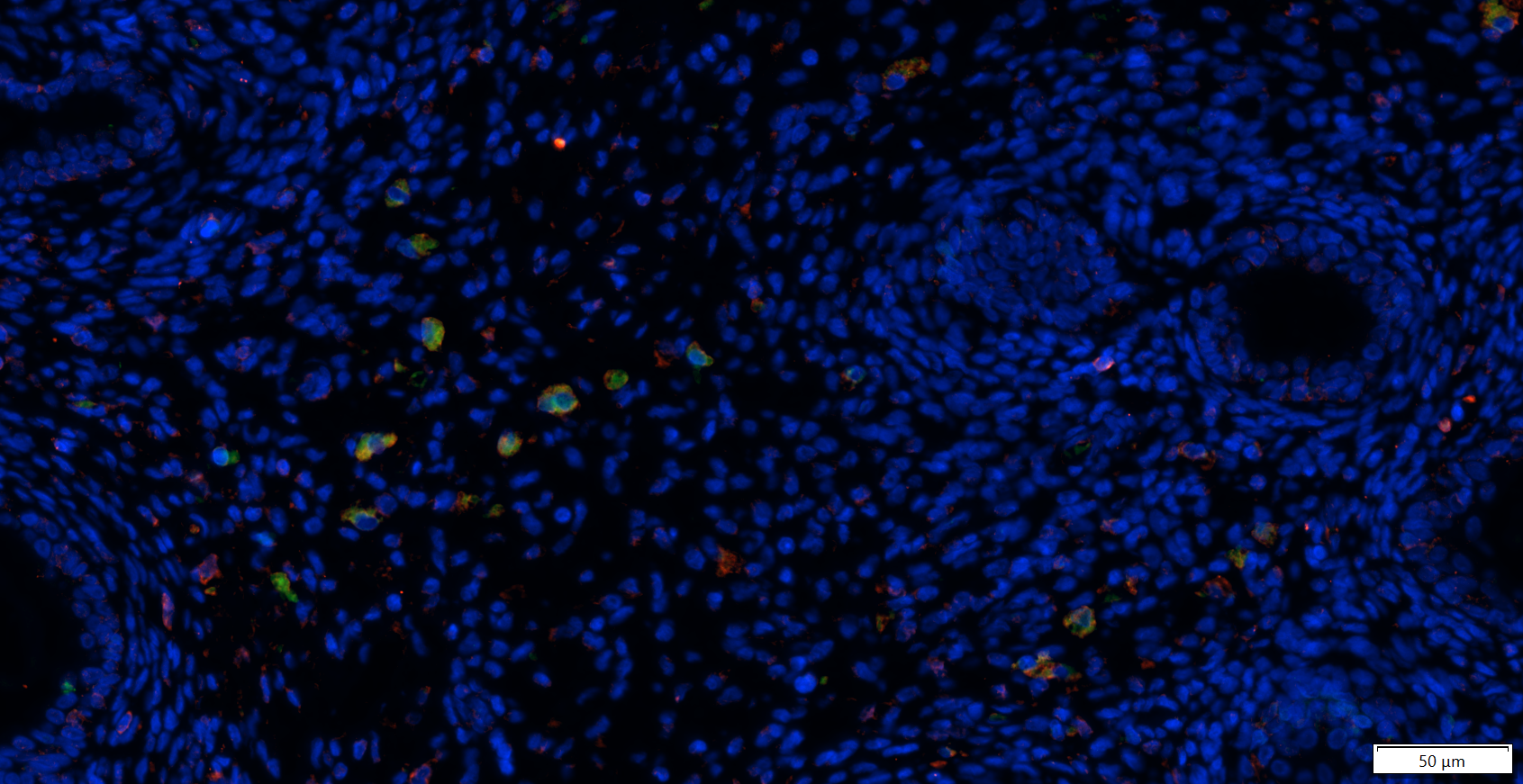

Supplement: S1 Data — (ZIP) [file pone.0309910.s001.zip › choose IF/fig2 B I 7C163.tif]

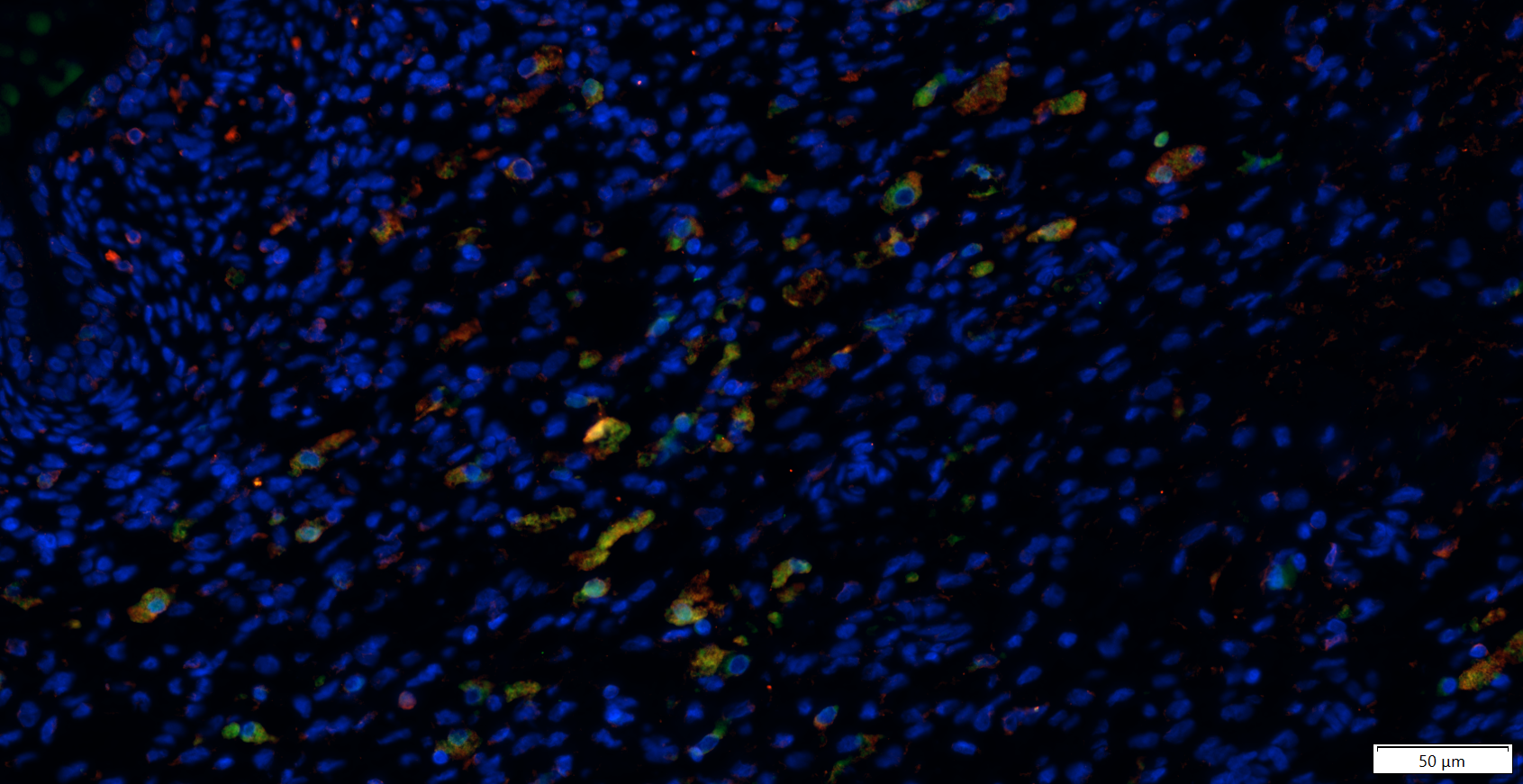

Supplement: S1 Data — (ZIP) [file pone.0309910.s001.zip › choose IF/fig2 B II 7B163.tif]

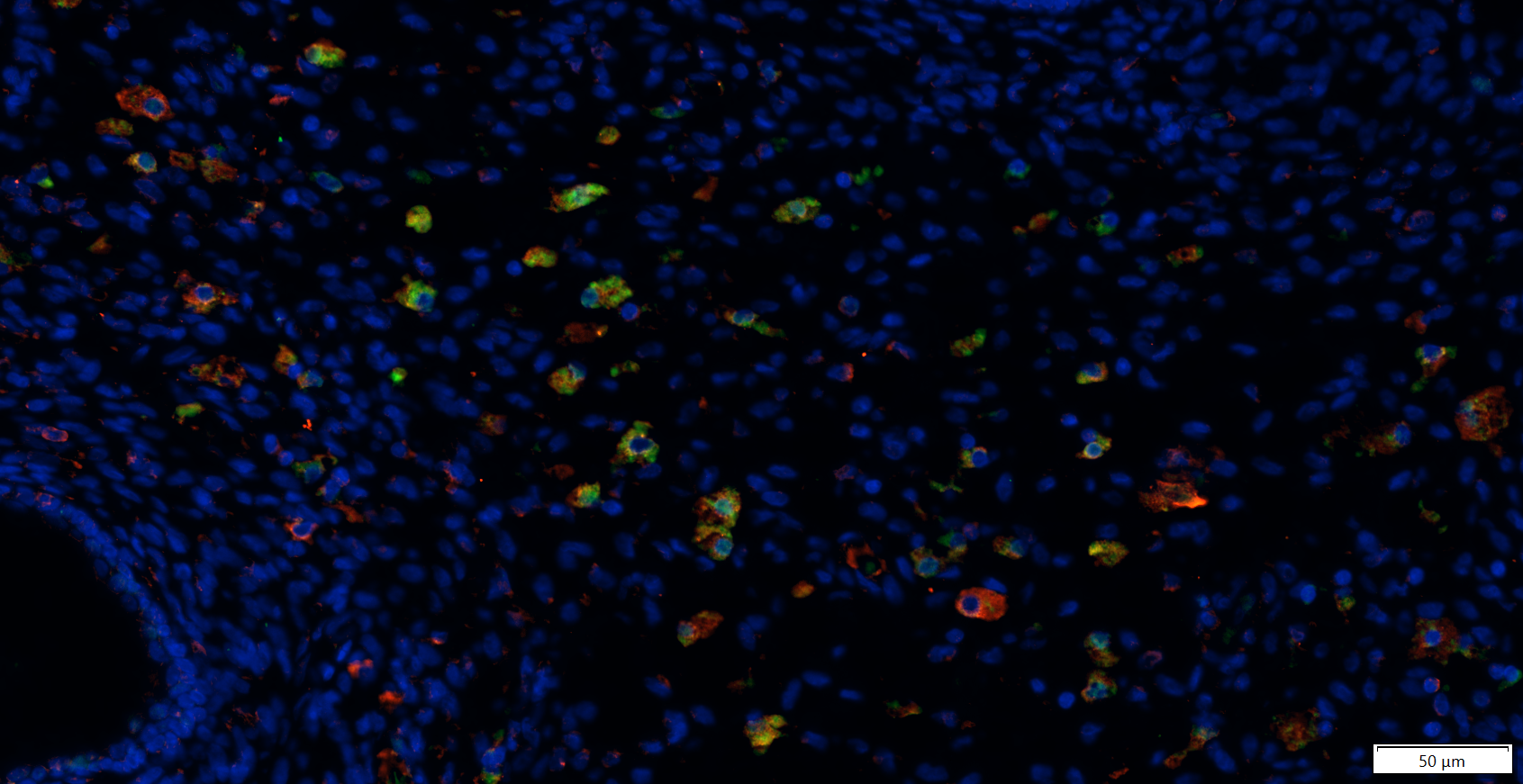

Supplement: S1 Data — (ZIP) [file pone.0309910.s001.zip › choose IF/fig2 B III 7A163.tif]

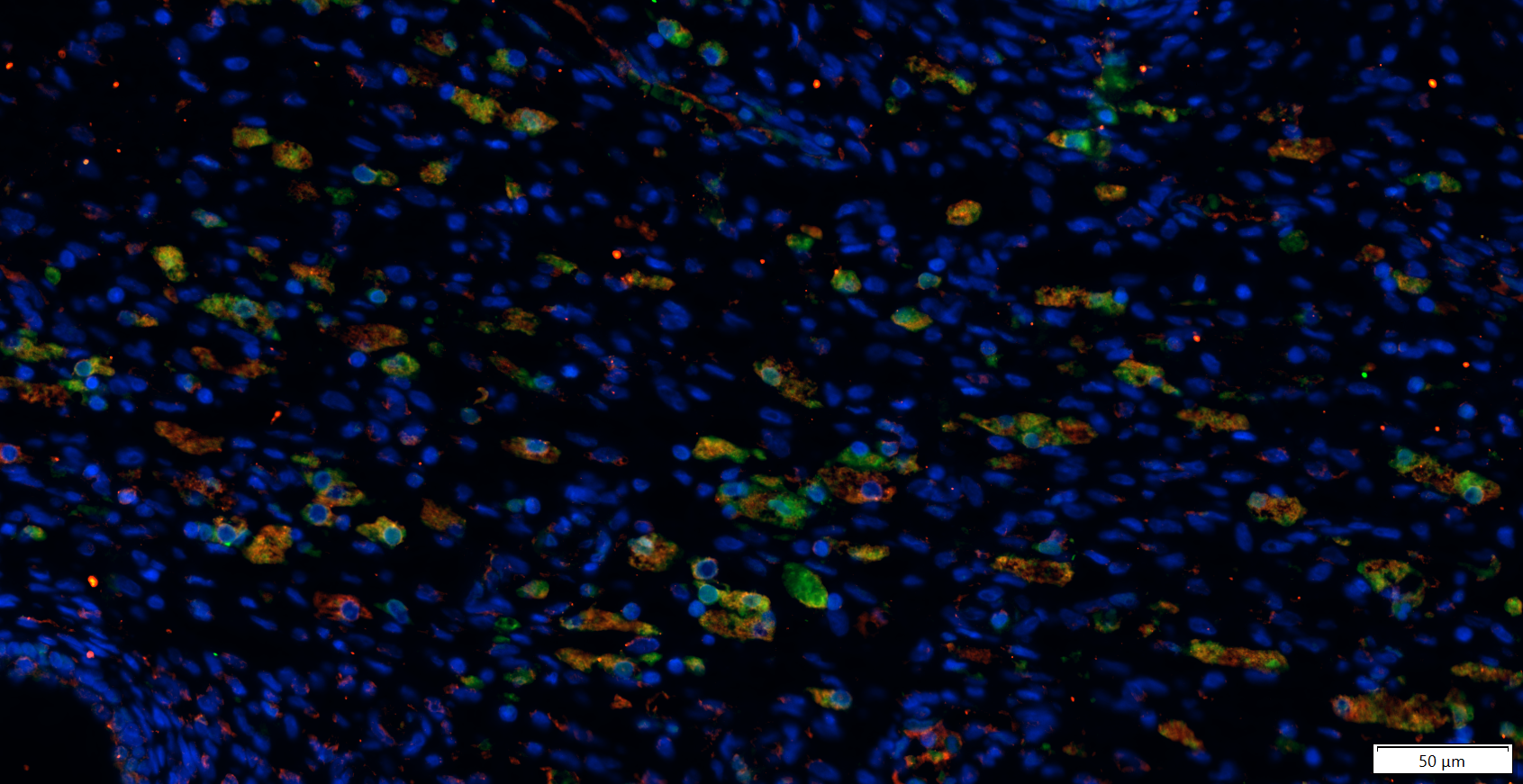

Supplement: S1 Data — (ZIP) [file pone.0309910.s001.zip › choose IF/fig2 B IV 7G163.tif]

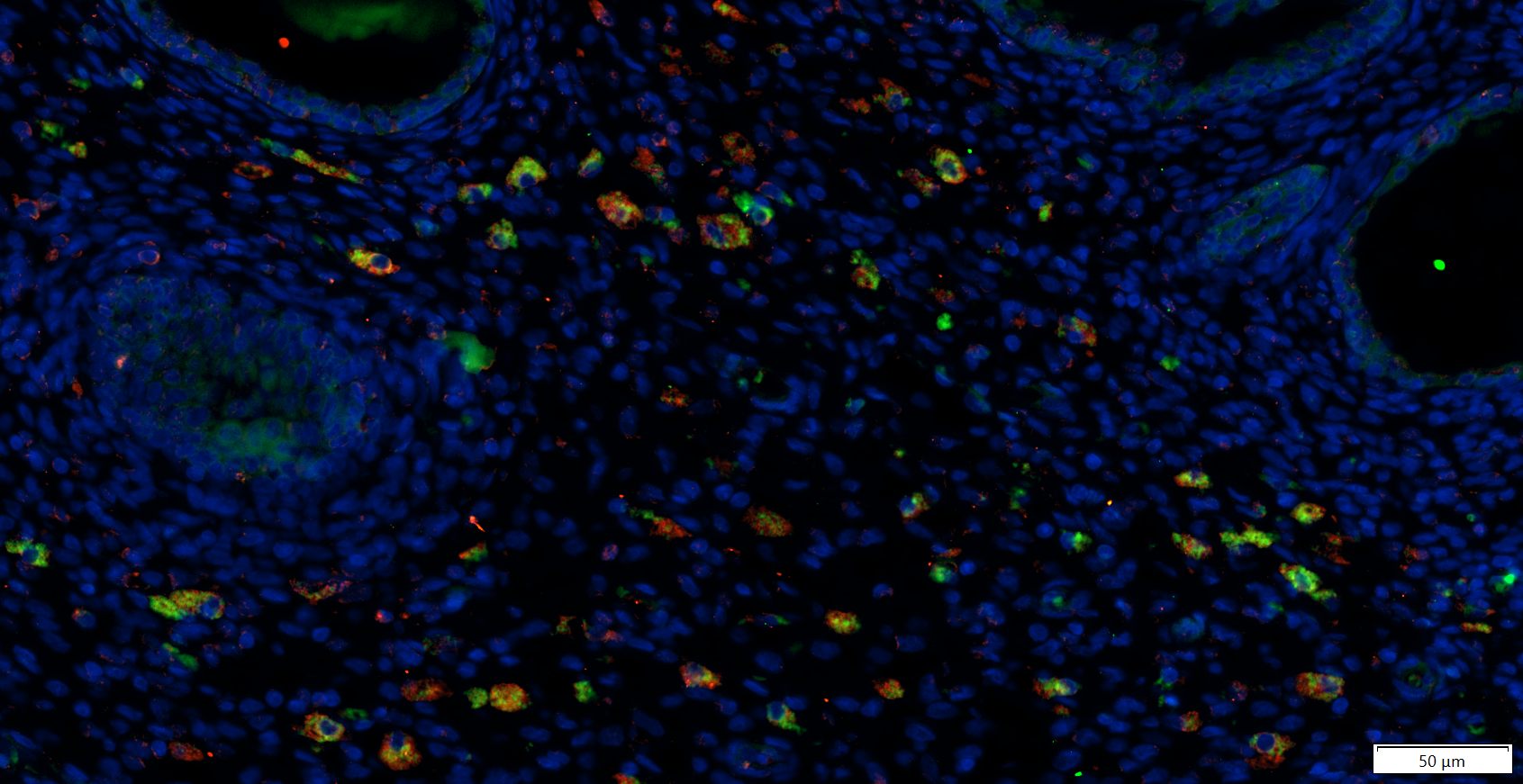

Supplement: S1 Data — (ZIP) [file pone.0309910.s001.zip › choose IF/fig2 C I 43 B 86.tif]

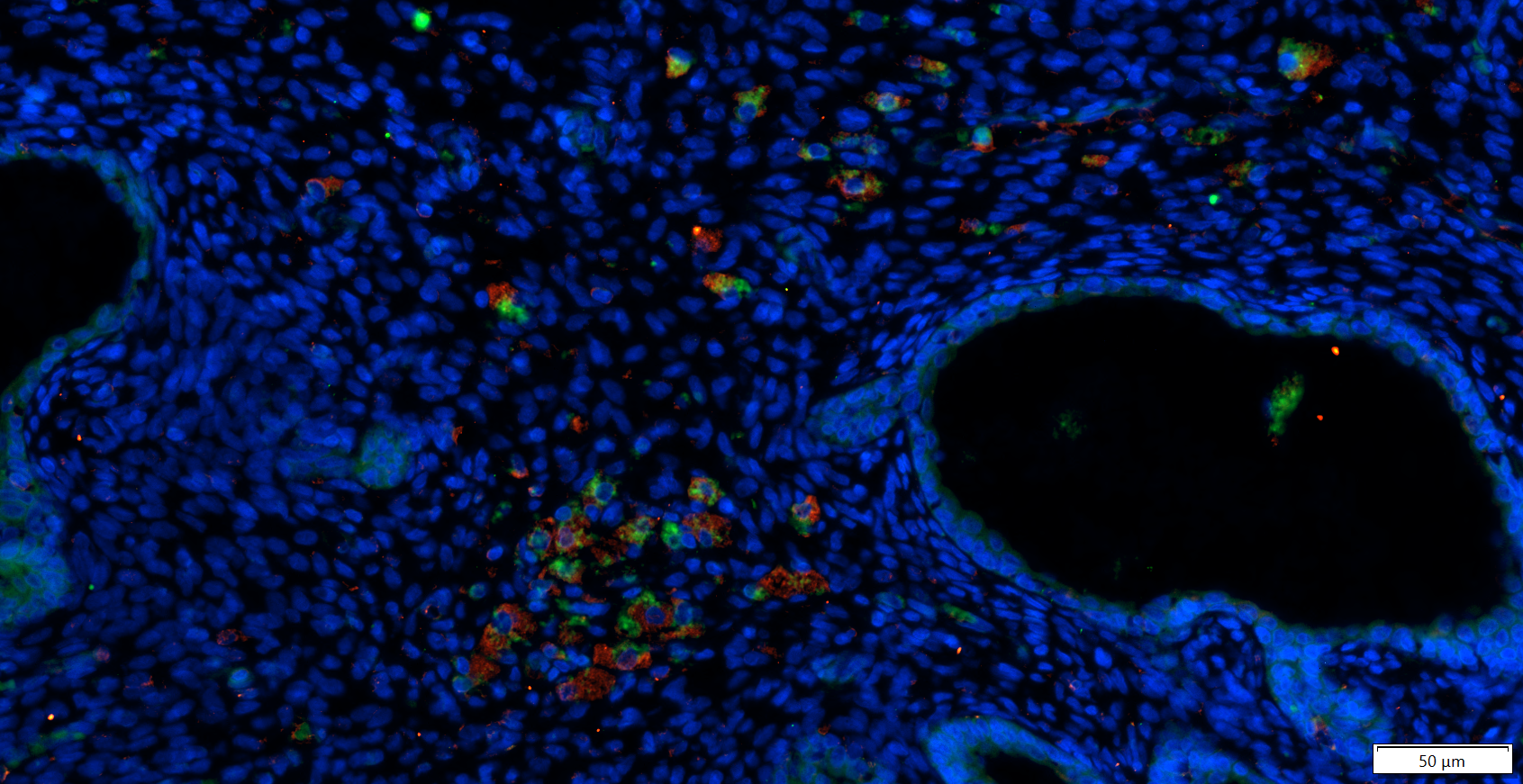

Supplement: S1 Data — (ZIP) [file pone.0309910.s001.zip › choose IF/fig2 C II 43 F 86.tif]

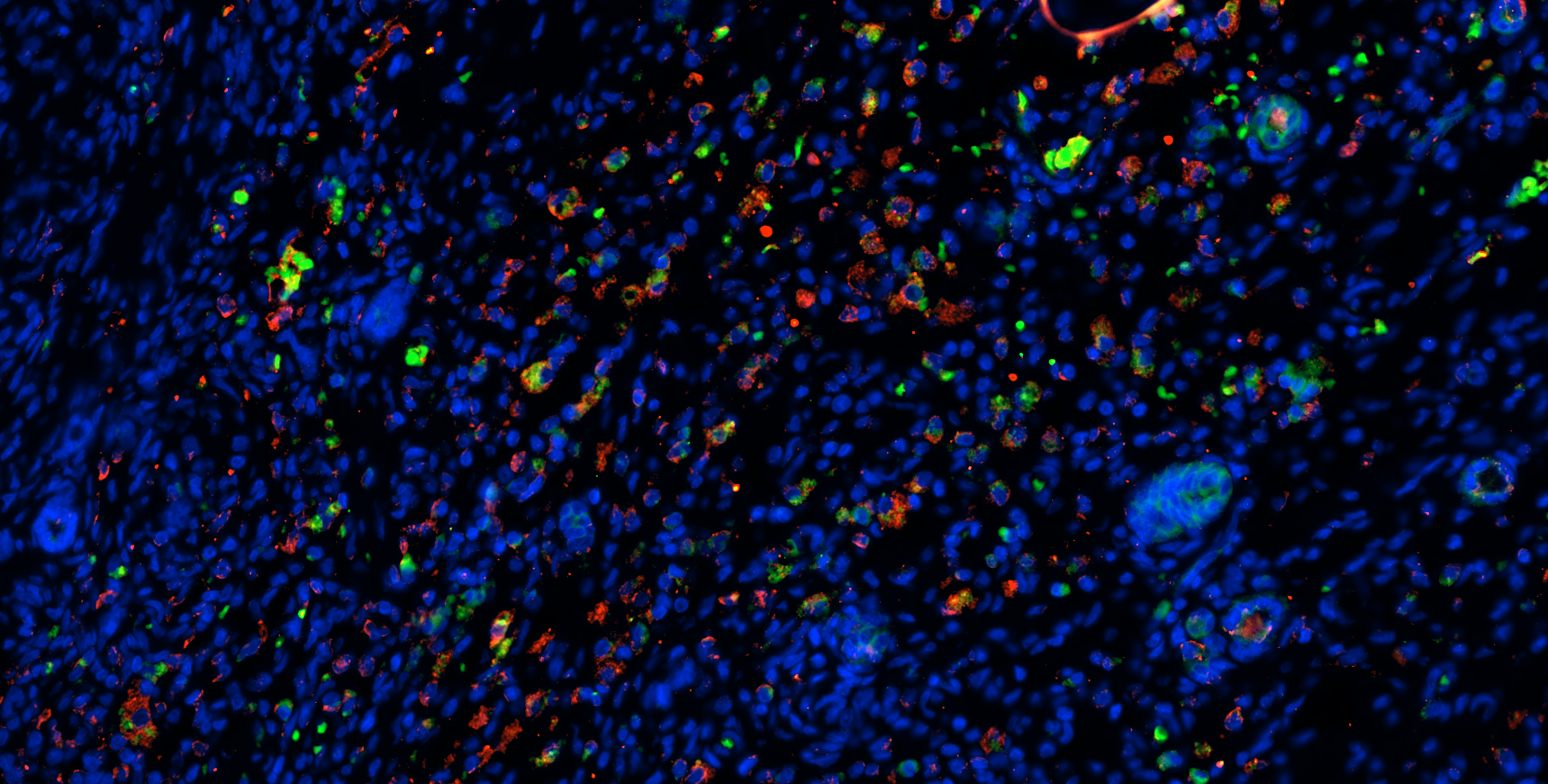

Supplement: S1 Data — (ZIP) [file pone.0309910.s001.zip › choose IF/fig2 C III 39 a.tif]

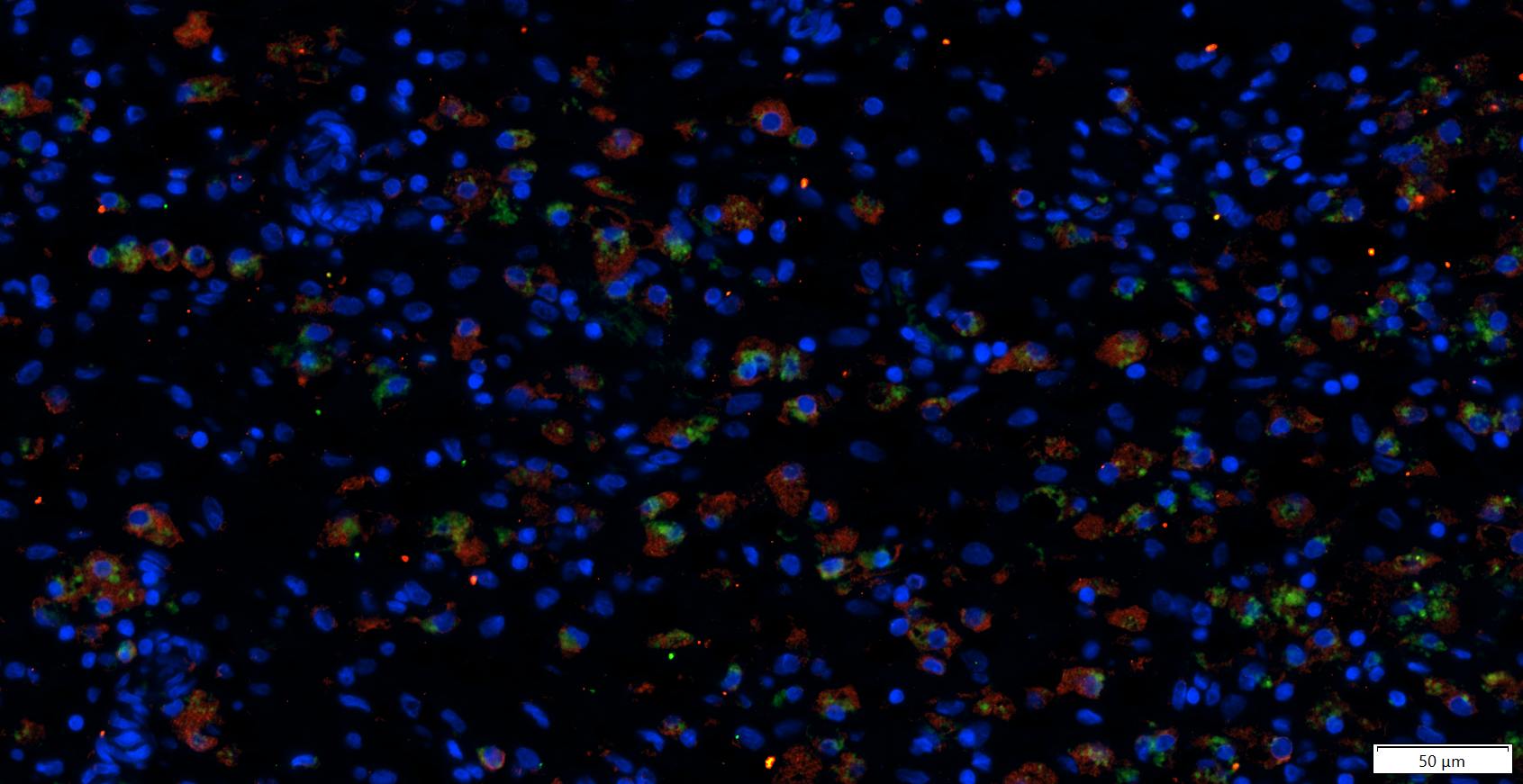

Supplement: S1 Data — (ZIP) [file pone.0309910.s001.zip › choose IF/fig2 C IV 43 C 86.tif]

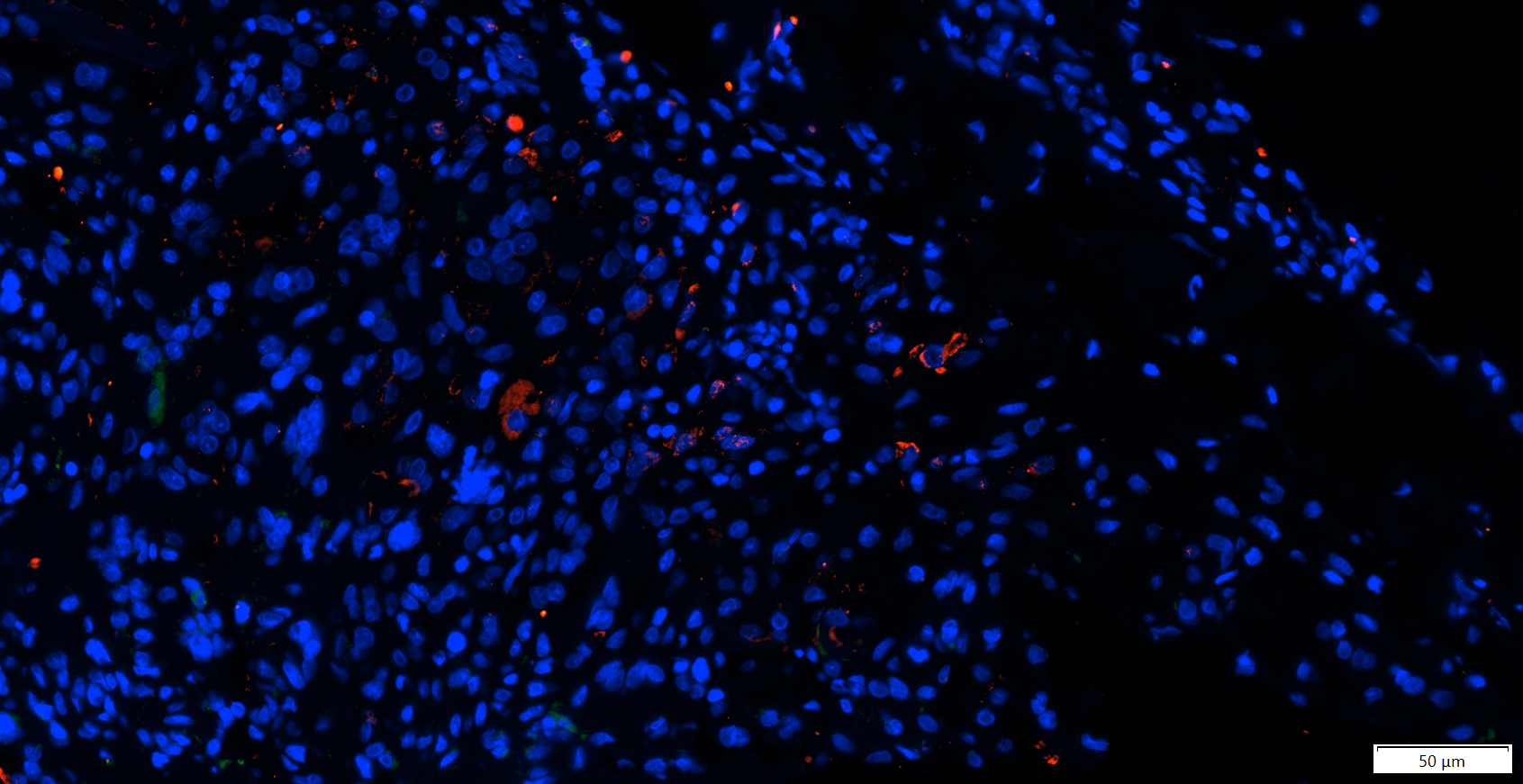

Supplement: S1 Data — (ZIP) [file pone.0309910.s001.zip › choose IF/fig2 D II 13A163 A1.tif]

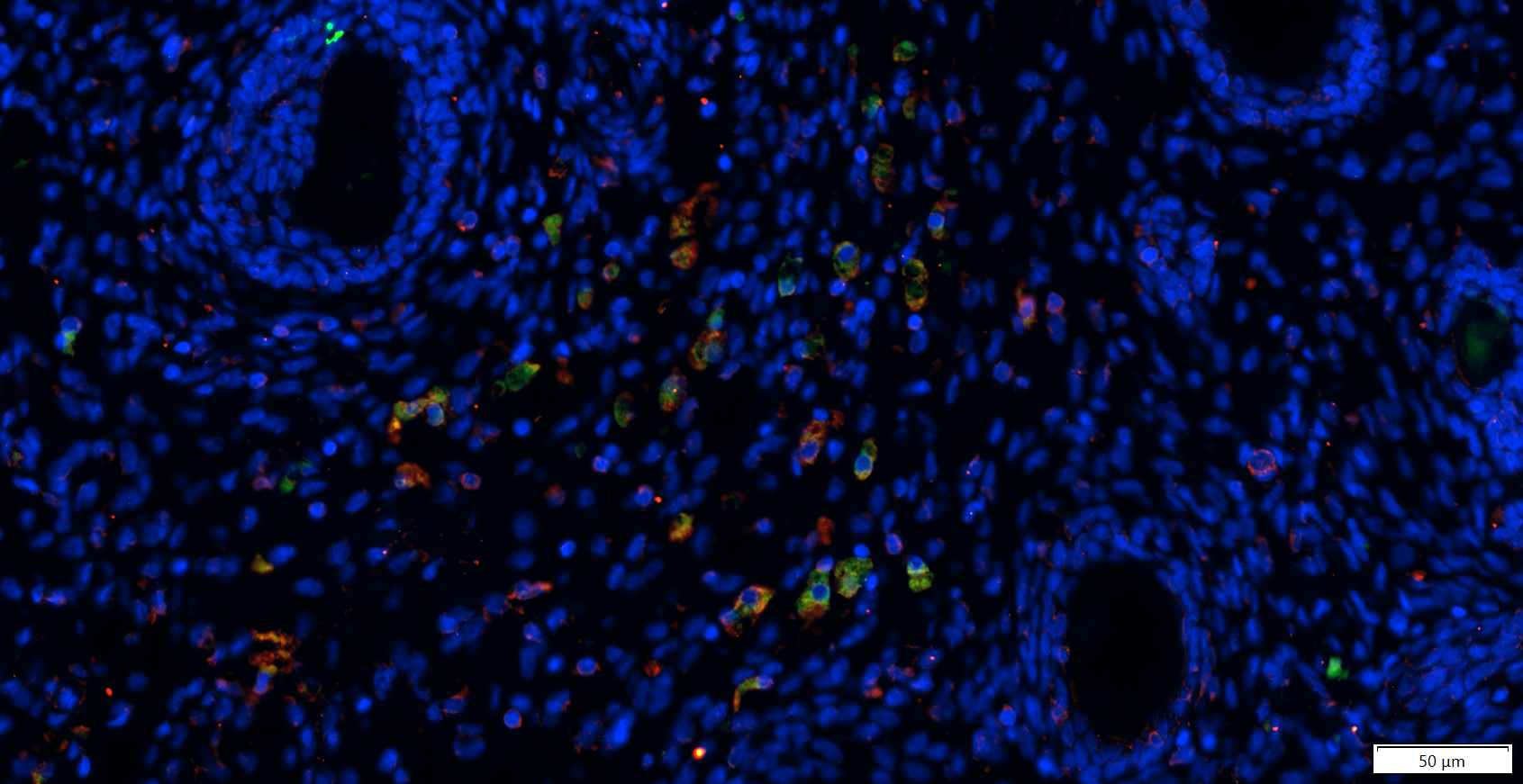

Supplement: S1 Data — (ZIP) [file pone.0309910.s001.zip › choose IF/fig2 D III 7H163.tif]

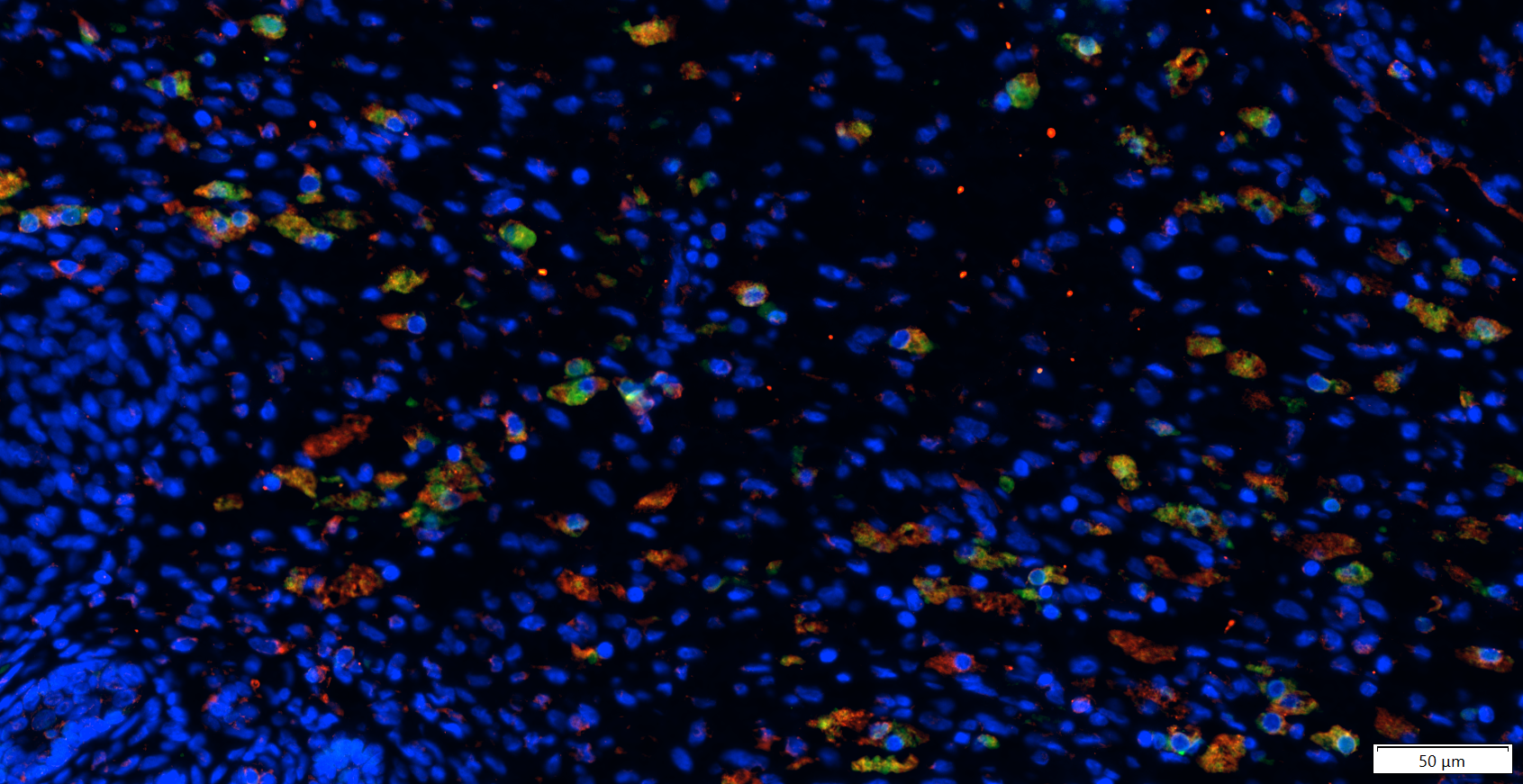

Supplement: S1 Data — (ZIP) [file pone.0309910.s001.zip › choose IF/fig2 D IV 7F163.tif]

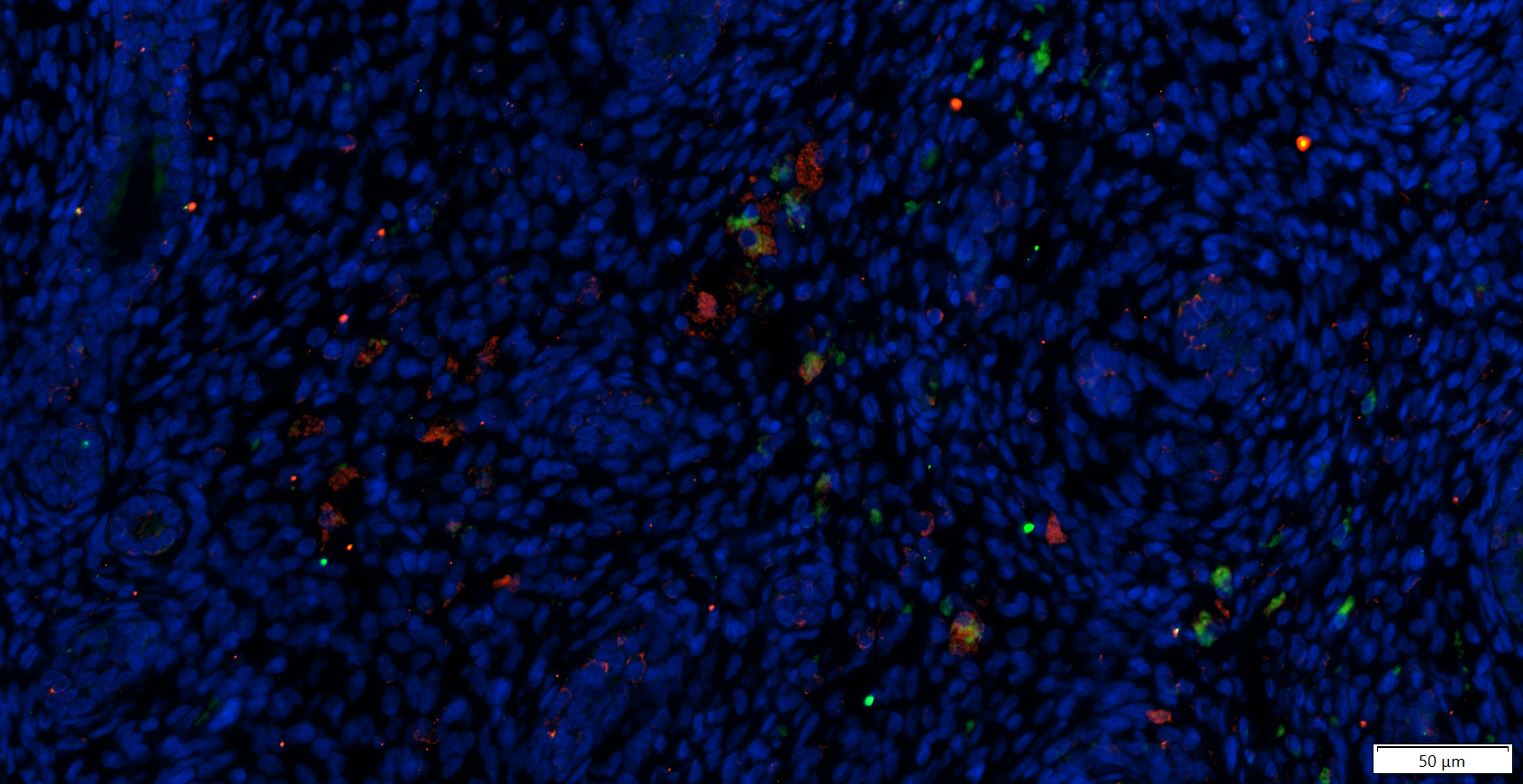

Supplement: S1 Data — (ZIP) [file pone.0309910.s001.zip › choose IF/fig2 E II 43 D 86.tif]

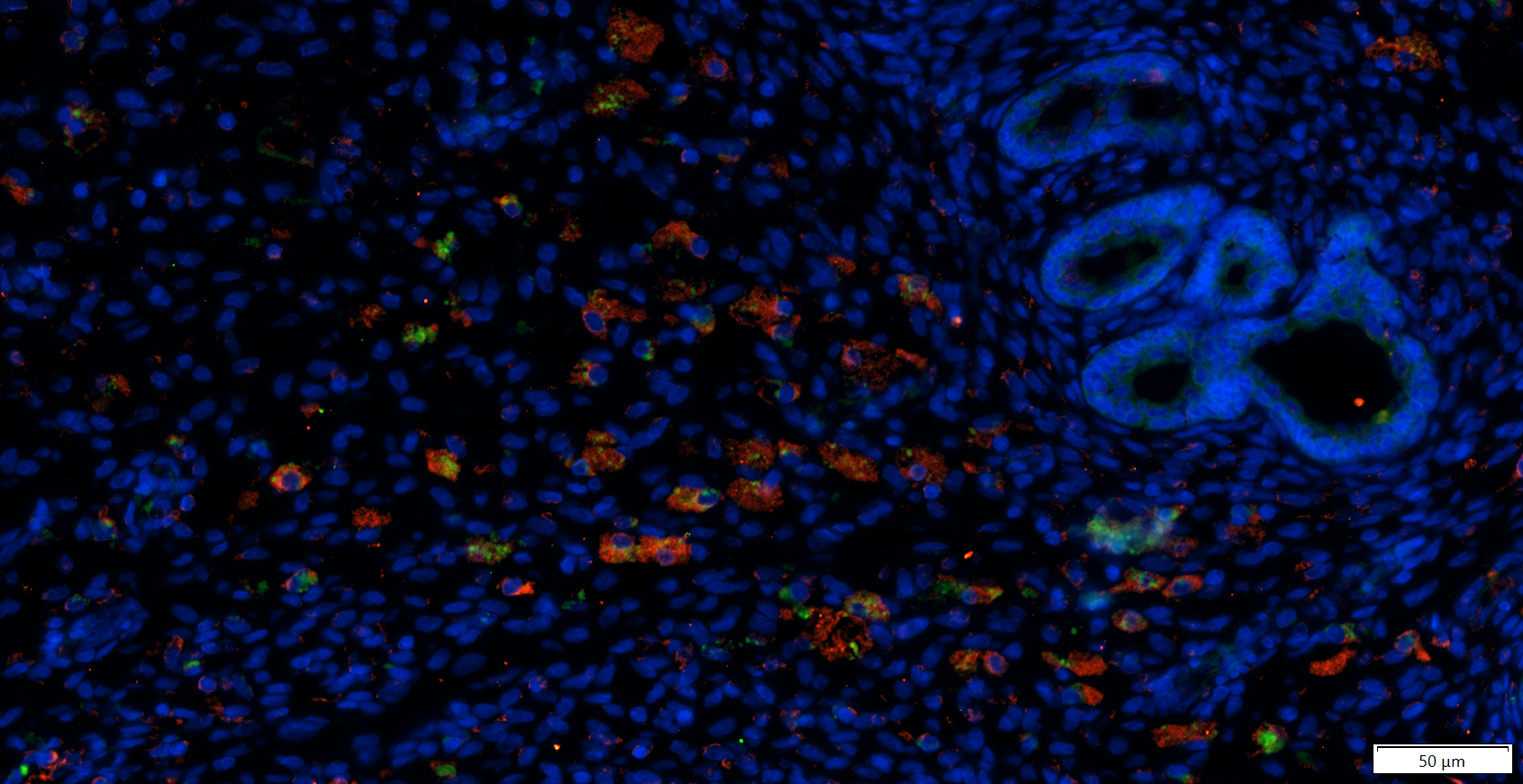

Supplement: S1 Data — (ZIP) [file pone.0309910.s001.zip › choose IF/fig2 E III 43 A 86.tif]

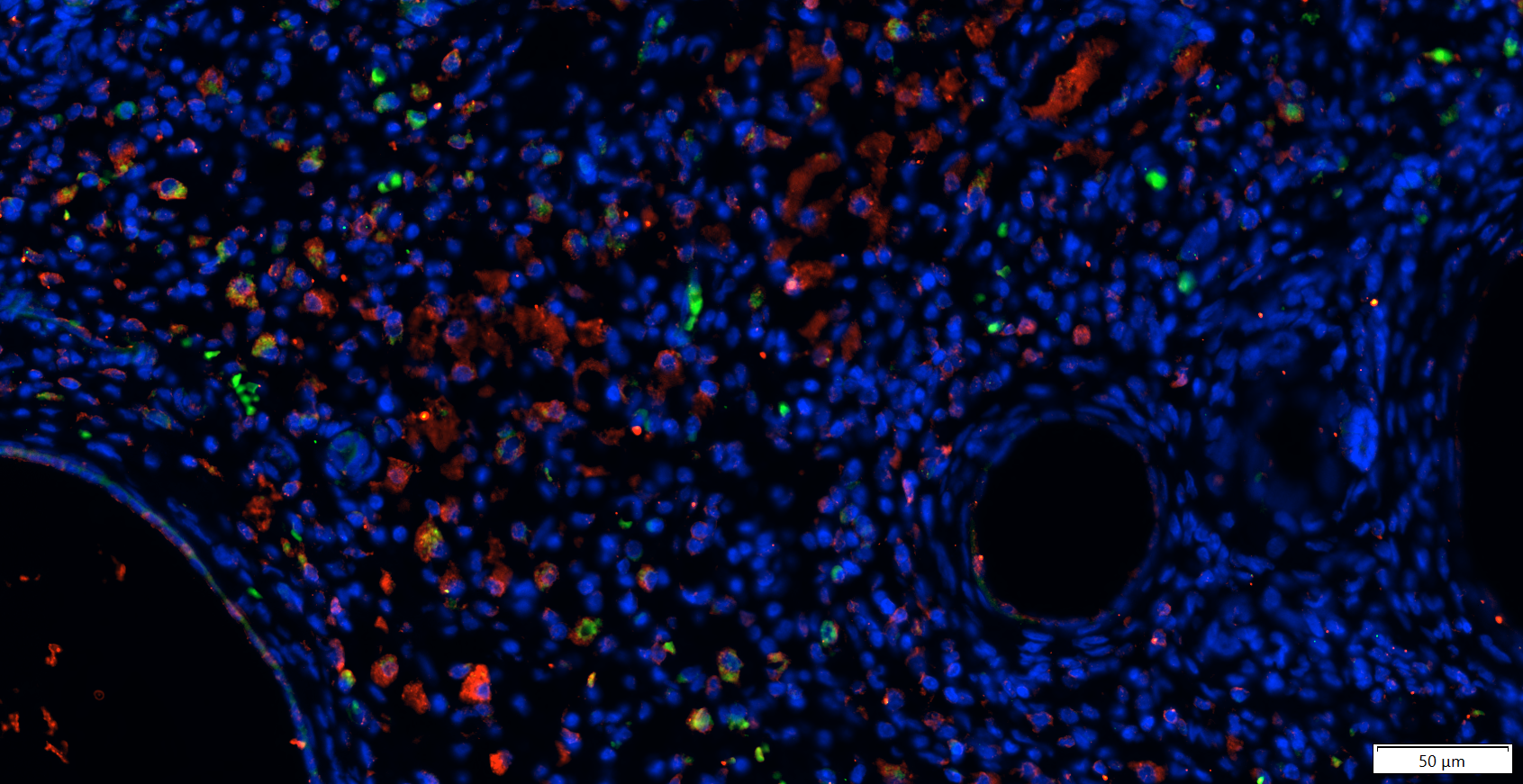

Supplement: S1 Data — (ZIP) [file pone.0309910.s001.zip › choose IF/fig2 E IV 39D86.tif]

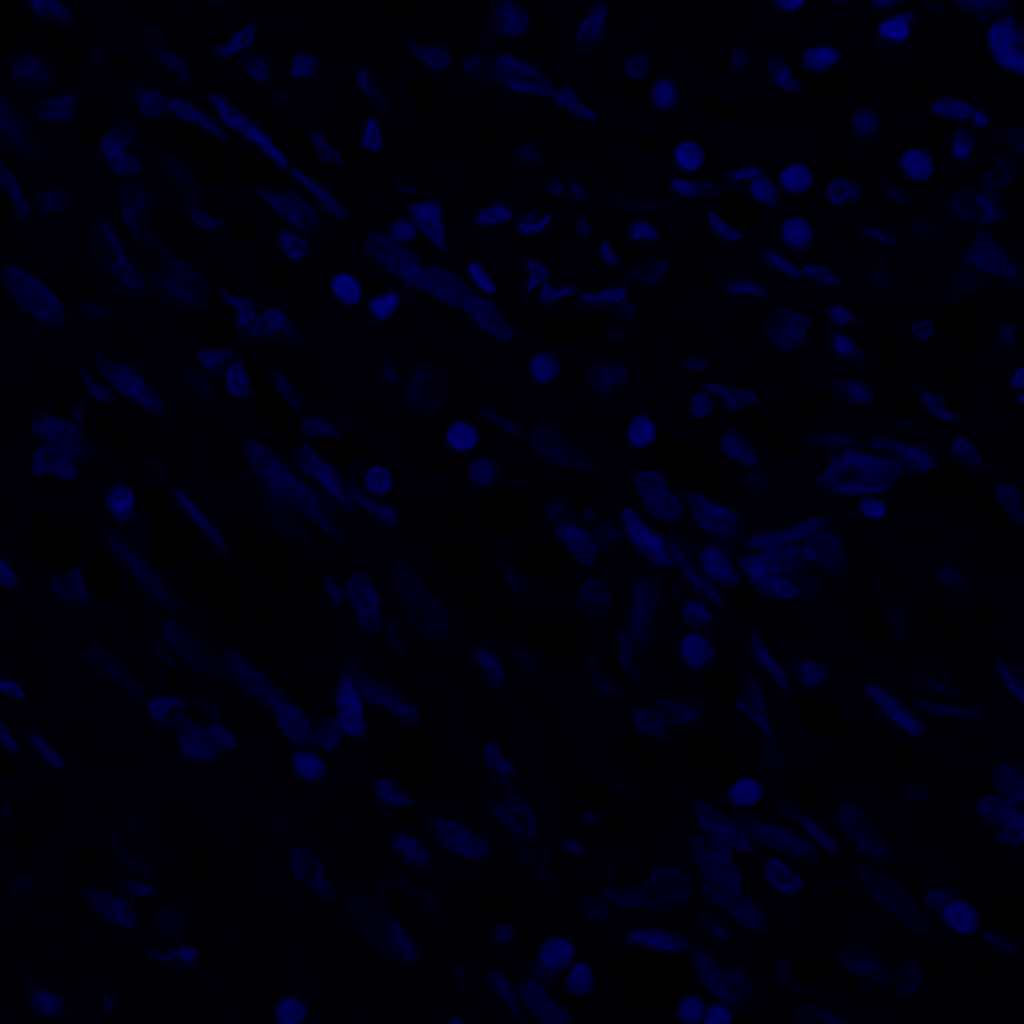

Supplement: S1 Data — (ZIP) [file pone.0309910.s001.zip › choose IF/fig2 a m1c1.tif]

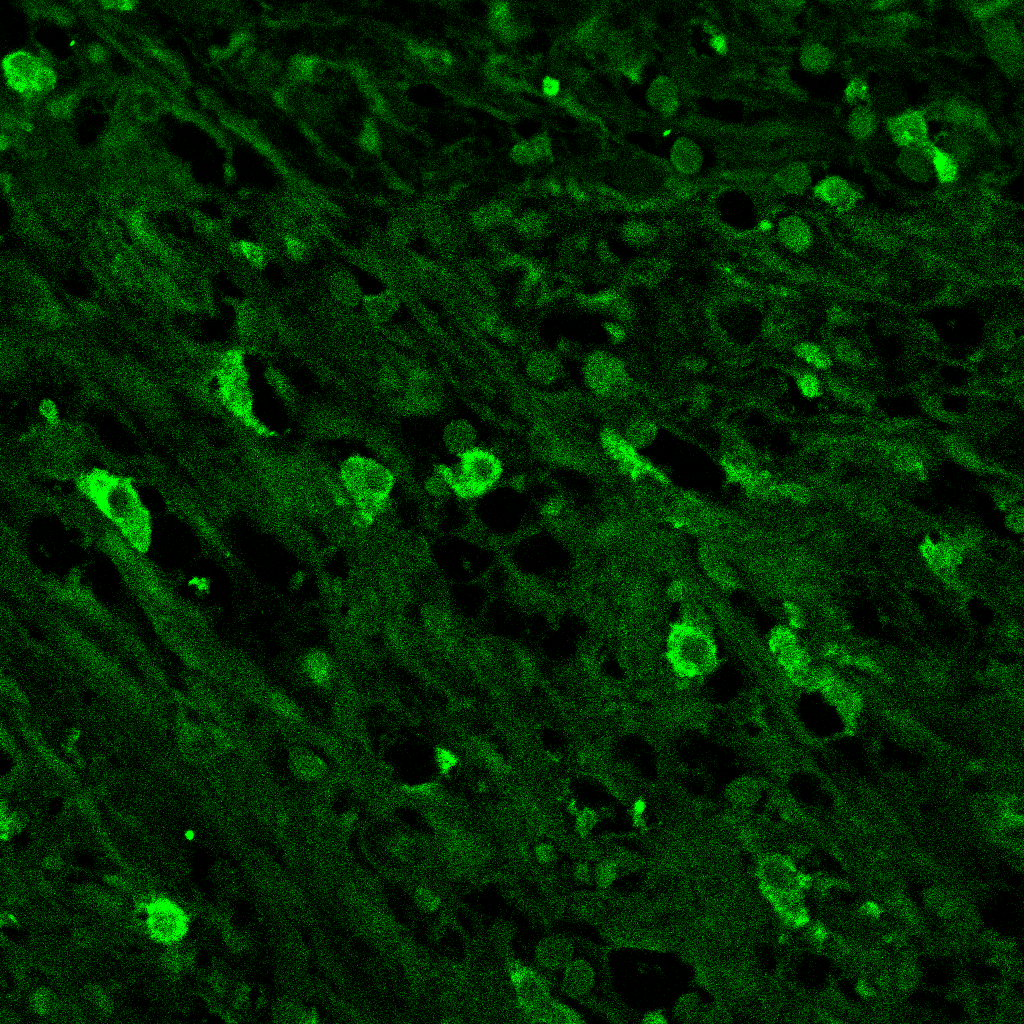

Supplement: S1 Data — (ZIP) [file pone.0309910.s001.zip › choose IF/fig2 a m1c2.tif]

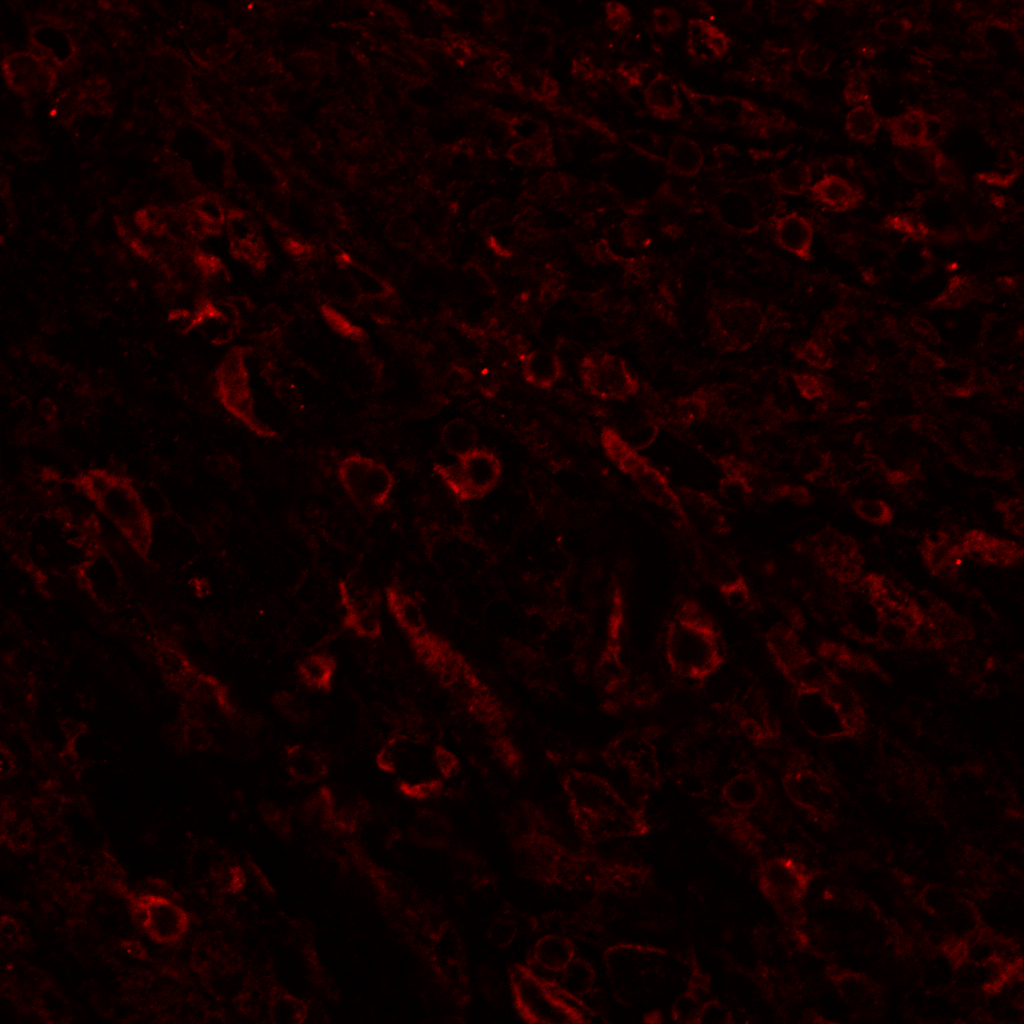

Supplement: S1 Data — (ZIP) [file pone.0309910.s001.zip › choose IF/fig2 a m1c3.tif]

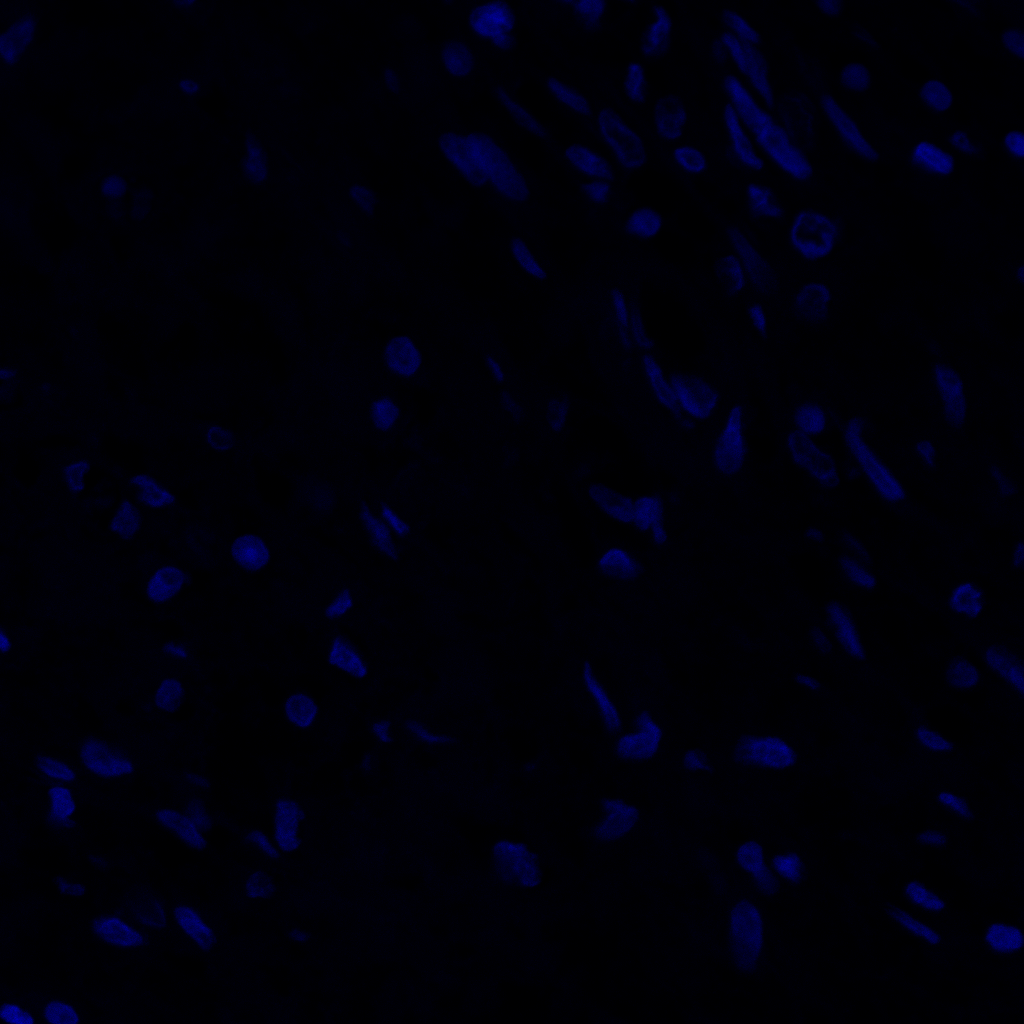

Supplement: S1 Data — (ZIP) [file pone.0309910.s001.zip › choose IF/fig2 a m2c1.tif]

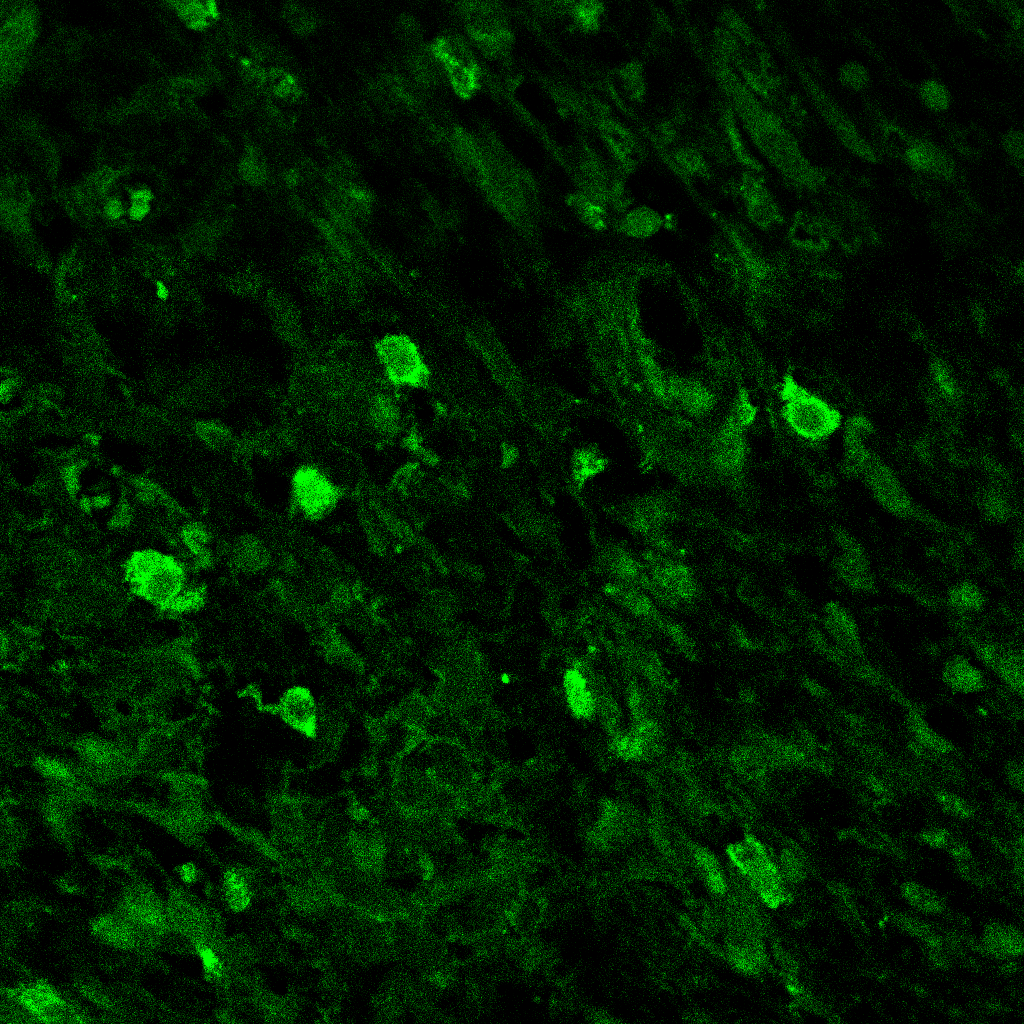

Supplement: S1 Data — (ZIP) [file pone.0309910.s001.zip › choose IF/fig2 a m2c2.tif]

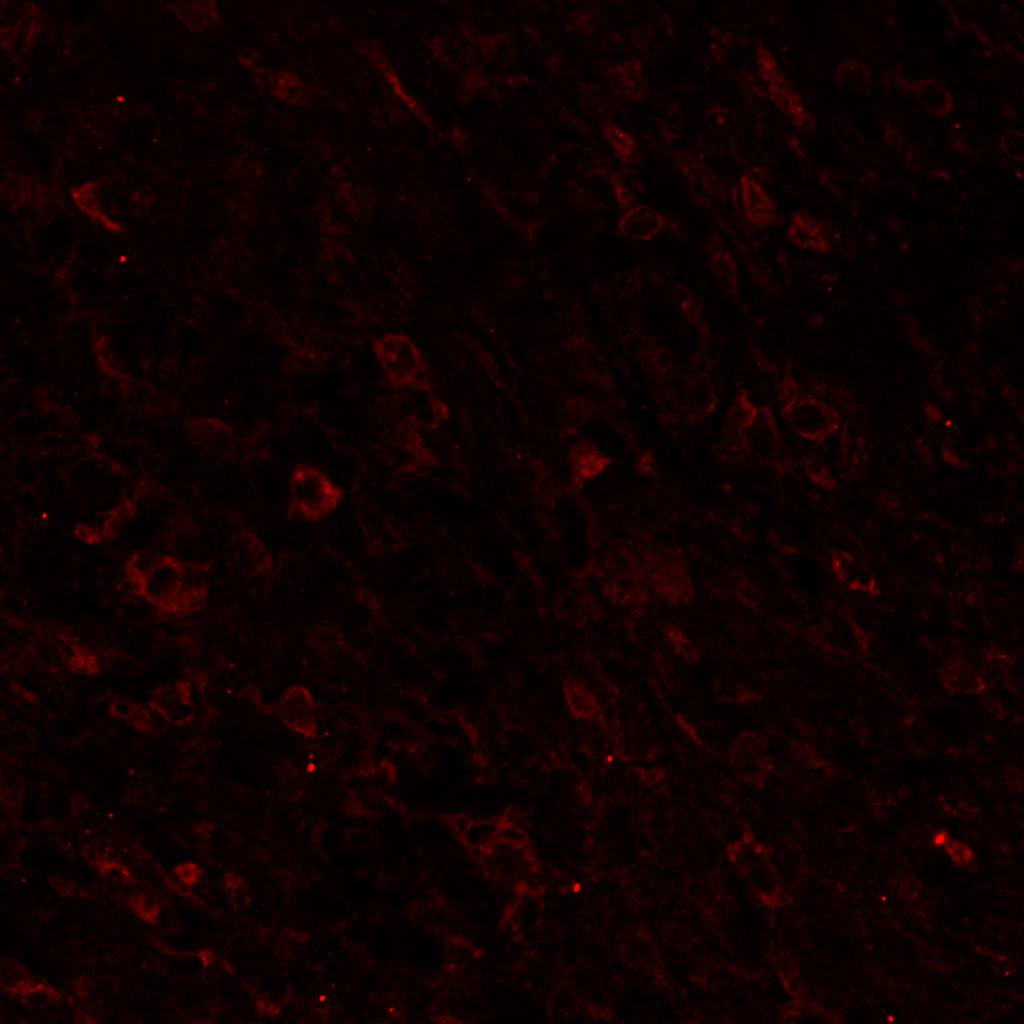

Supplement: S1 Data — (ZIP) [file pone.0309910.s001.zip › choose IF/fig2 a m2c3.tif]

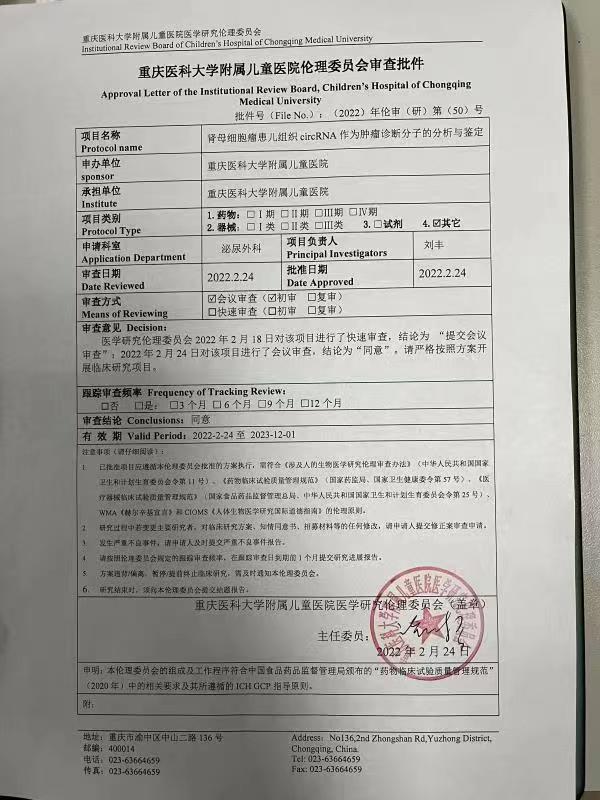

Supplement: S2 Data — (ZIP) [file pone.0309910.s002.zip › the minimal data set/IRB-2022-50.jpg]
