# Supplementary material for: Recruitment and polarization typing of tumor-associated macrophages is associated with tumor progression and poor prognosis in Wilms tumor patients
Source: PLoS One. 2024 Nov 12;19(11):e0309910. doi: 10.1371/journal.pone.0309910 (PMC11556688; doi:10.1371/journal.pone.0309910)
Supplement: S2 Data — (ZIP) [file pone.0309910.s002.zip › the minimal data set/raw wb tiff.zip!/raw wb.pdf]

Tumor 1-3

CD163

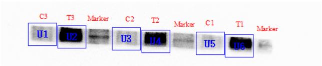

2023-02-26 07hr 11min 46sec 1-3 CD163.scn

CD86

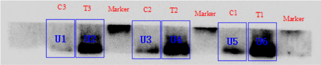

2023-02-26 07hr 08min 51sec 1-3 CD86.scn

GAPDH

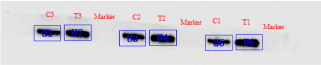

2023-02-26 06hr 48min 15sec 1-3 b-Actin.scn

Tumor 4-6

CD163

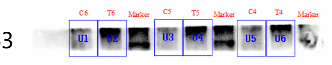

2023-02-26 06hr 31min 57sec 4-6 CD163.scn

CD86

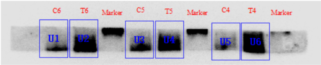

2023-02-26 07hr 07min 43sec 4-6 CD86.scn

GAPDH

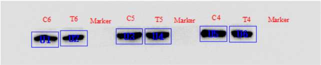

2023-02-26 06hr 26min 40sec 4-6 b-Actin.scn

Tumor 7-8

CD163

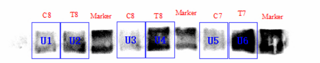

2023-02-27 06hr 17min 09sec 7-8 CD163.scn

CD86

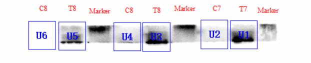

2023-02-27 06hr 23min 52sec 7-8 CD86.scn

GAPDH

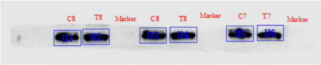

2023-02-27 06hr 54min 50sec 7-8 b-Actin.scn
